# Switching harmful visceral fat to beneficial energy combustion improves metabolic dysfunctions

**DOI:** 10.1172/jci.insight.89044

**Published:** 2017-02-23

**Authors:** Xiaoyan Yang, Wenhai Sui, Meng Zhang, Mei Dong, Sharon Lim, Takahiro Seki, Ziheng Guo, Carina Fischer, Huixia Lu, Cheng Zhang, Jianmin Yang, Meng Zhang, Yangang Wang, Caixia Cao, Yanyan Gao, Xingguo Zhao, Meili Sun, Yuping Sun, Rujie Zhuang, Nilesh J. Samani, Yun Zhang, Yihai Cao

**Affiliations:** 1 The Key Laboratory of Cardiovascular Remodeling and Function Research, Chinese Ministry of Education and Chinese Ministry of Public Health, Shandong University Qilu Hospital, Jinan, Shandong, China.; 2 Department of Microbiology, Tumor and Cell Biology, Karolinska Institutet, Stockholm, Sweden.; 3 Department of Cardiology, Beijing Chaoyang Hospital, Capital Medical University, Beijing, China.; 4 West China School of Medicine, Sichuan University, Chengdu, China.; 5 Department of Endocrinology and Metabolism, Affiliated Hospital of Qingdao University, Qingdao, China.; 6 Department of Otolaryngology, Shandong University Qilu Hospital, Jinan, Shandong, China.; 7 Department of Oncology, Jinan Central Hospital, Shandong University, Jinan, Shandong, China.; 8 The TCM Hospital of Zhejiang Province, Hangzhou, Zhejiang, China.; 9 Department of Cardiovascular Sciences, University of Leicester and NIHR Leicester Cardiovascular Biomedical Research Unit, Glenfield Hospital, Leicester, United Kingdom.

## Abstract

Visceral fat is considered the genuine and harmful white adipose tissue (WAT) that is associated to development of metabolic disorders, cardiovascular disease, and cancer. Here, we present a new concept to turn the harmful visceral fat into a beneficial energy consumption depot, which is beneficial for improvement of metabolic dysfunctions in obese mice. We show that low temperature–dependent browning of visceral fat caused decreased adipose weight, total body weight, and body mass index, despite increased food intake. In high-fat diet–fed mice, low temperature exposure improved browning of visceral fat, global metabolism via nonshivering thermogenesis, insulin sensitivity, and hepatic steatosis. Genome-wide expression profiling showed upregulation of WAT browning–related genes including *Cidea* and *Dio2*. Conversely, *Prdm16* was unchanged in healthy mice or was downregulated in obese mice. Surgical removal of visceral fat and genetic knockdown of UCP1 in epididymal fat largely ablated low temperature–increased global thermogenesis and resulted in the death of most mice. Thus, browning of visceral fat may be a compensatory heating mechanism that could provide a novel therapeutic strategy for treating visceral fat–associated obesity and diabetes.

## Introduction

Obesity is an epidemic predisease condition that contributes to high incidences of type 2 diabetes, cardiovascular disease, inflammatory disorders, and certain cancers (1–5). S.c. obesity is relatively harmless, but visceral fat adiposity is highly associated with high incidence of type 2 diabetes and mortality, even in people with normal body index (6, 7). Thus, distinguishing the adiposity location may predict the prevalence of metabolic diseases.

In support of this notion, a series of studies showed the existence of a number of molecular signatures in brown adipose tissue (BAT), beige/brite/browning adipose tissue, and “genuine” white adipose tissue (WAT) (8–13). Furthermore, white and brown adipocytes are not derived from the same precursor cells, but rather, BAT adipocytes arise from a Myf5^+^ myocyte-like lineage, although adiomyocytes could be a common precursor (14–17). The transcription factor PRDM16 defines browning adipocytes in s.c. WAT (7). With selective deletion of PRDM16 in adipocytes without affecting classical BAT function, s.c. WAT loses its browning ability but acquires the morphological and functional characteristics of visceral fat (7). Functionally, mice lacking functional adipocyte PRDM16 show obesity, liver steatosis, and insulin resistance. Thus, PRDM16 is the key determinant for beige adipocyte development and regulation of global metabolism (7).

Among all browning agents, cold exposure–stimulated sympathetic activation is probably the most physiologically relevant WAT browning stimulus (18, 19). Exposure of rodents to 4°C induces a browning phenotype of s.c. WAT, along with increased uncoupling protein 1–dependent (UCP1-dependent) heat production (20–23). Unlike s.c. WAT, this same cold condition cannot significantly induce browning of visceral fat including epididymal and mesenteric fat depots (7, 21–23). However, it has been shown that visceral fat can begin browning under certain circumstances. For example, retinaldehyde dehydrogenase 1–deficient mice show browning of visceral fat (24). Additionally, WAT browning is also controlled by genetic factors (25). From these and other findings, s.c. WAT and visceral WAT are believed to be functionally different fat depots. However, constant 4°C rarely exists in any given natural environment, and relentless variations of temperature occur. To the best of our knowledge, no study has exposed experimental mice to freezing low temperatures to investigate the browning activation of various adipose depots.

In this work, we exposed mice to physiologically tolerable freezing temperature — not just the classical 4°C, widely used to study BAT activation and browning of s.c. WAT (19, 20, 22, 23) — to induce browning of visceral fat. Visceral fat browning in obese mice under freezing low temperature had a global functional impact on improving thermogenic metabolism, insulin sensitivity, and liver steatosis. Visceral fat may serve as a reserve mechanism of heat production in defending against severe low temperature for survival in mice. Moreover, human preadipocytes from visceral WAT have the bona fide capacity to differentiate UCP1-positive browning adipocytes. If the same mechanism exists in humans, our findings may provide a new therapeutic option for visceral fat obesity and its related metabolic diseases.

## Results

### Impact of low temperature on food intake, body weight, and core body temperature.

**Figure 1:**
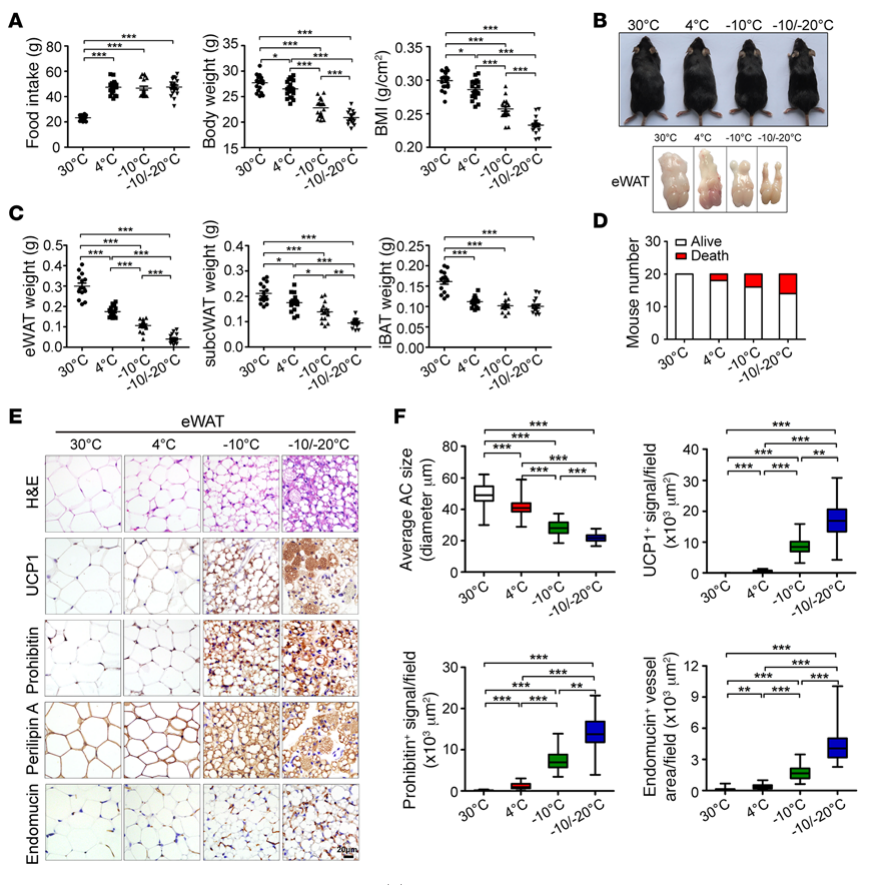
Food intake, body weight, BMI, browning of visceral WAT. (**A**) Food intake per week, body weight, and BMI with exposure to various temperatures (*n* = 20 mice per group, data represent mean ± SEM, one-way ANOVA). (**B**) Mouse morphology and epididymal WAT (eWAT) morphology. (**C**) Fat mass of eWAT, s.c. WAT, and interscapular brown adipose tissue (iBAT) (*n* = 15 mice per group, data represent mean ± SEM, one-way ANOVA). (**D**) Contingent survival and death of mice exposed to various temperatures (*n* = 20 mice per group). (**E**) H&E, UCP1, prohibitin, perilipin A, and endomucin staining of eWAT. Scale bar: 20 μm. (**F**) Quantification of adipocyte (AC) size and UCP1-, prohibitin-, and endomucin-positive signals of eWAT (50 random fields from 10 mice in each group). **P < 0.01; **P < 0.01; ***P < 0.001, one-way ANOVA. Box-and-whisker plots show median (line within box), upper and lower quartile (bounds of box), and minimum and maximum values (bars).

To study the effect of physiologically tolerable low temperature on mouse food intake and body weight, we exposed C57BL/6J mice to various low temperatures, including 4°C, –10°C, and –10°C/–20°C. The thermoneutral temperature 30°C was used as a control. During cold exposure at 4°C, –10°C, and –10°C/–20°C, food intake was markedly increased, with no significant differences among the three cold-exposed groups (Figure 1A), but the total body weight of the three groups of animals was significantly decreased with reducing environmental temperature relative to the thermoneutral condition; consequently, the mice exposed to –10°C/–20°C showed the lowest body weight, which was significantly lower than with 4°C and –10°C (Figure 1, A and B). Similar to body weight loss, BMI was progressively reduced with temperature reduction (Figure 1A). The lean body was also decreased (Supplemental Figure 1A; supplemental material available online with this article; https://doi.org/10.1172/jci.insight.89044DS1). Under these low-temperature conditions, the core body temperature of mice remained unchanged (Supplemental Figure 1B). Thus, progressively reducing environmental temperature caused a food intake–independent reduction in body weight and BMI in mice. Additionally, physical activity was markedly reduced in extreme ambient low temperature groups relative to the 4°C group (Supplemental Figure 1C). By contrast, nesting materials were significantly increased in extreme low-temperature groups (Supplemental Figure 1C).

### Low temperature–dependent fat mass reduction and browning of visceral fat depot.

The total mass of epididymal WAT (eWAT), a “genuine” or “real” white fat depot, was markedly reduced with decreased environmental temperature. eWAT mass decreased approximately 80% with –10°C/–20°C compared with 30°C (Figure 1, B and C). Reduction in eWAT mass appeared to be dependent on reducing temperature, and eWAT weight was decreased more than 50% with –10°C/–20°C relative to 4°C (Figure 1C). Similar to eWAT, the s.c. WAT mass was reduced with lower temperature, although to a lesser extent than for eWAT (Figure 1C). In contrast to s.c. and visceral WATs, the total BAT weight at 4°C, –10°C, and –10°C/–20°C remained unchanged, although BAT masses of these groups were significantly lower than they were in thermoneutrality (Figure 1C). Along with decreasing temperature, increases of mouse mortality were also observed (Figure 1D).

Adipocytes under 30°C appeared to be larger and to be unilocular cells with cell nucleus located at the peripheral region (Figure 1E). With 4°C, the unilocular larger adipocytes of eWAT remained modestly decreased in size relative to 30°C (Figure 1E). However, with –10°C and –10°C/–20°C, the unilocular larger adipocytes appeared smaller, and multilocular cells contained a high density of intracellular organelles. Moreover, with –10°C and –10°C/–20°C, eWAT adipocytes expressed a high level of UCP1 protein, with the highest expression at –10°C/–20°C (Figure 1, E and F). The total mitochondrial content was higher in eWAT adipocytes with –10°C and –10°C/–20°C than with 30°C and 4°C. Other parameters, including adipocyte size as measured by perilipin A and endomucin-positive microvessel density, were proportionally altered, along with reducing temperature (Figure 1, E and F). Exposure to the classically 4°C cold environment did not substantially alter UCP1 and mitochondrial content in eWAT (Supplemental Figure 2A).

Histological and immunohistochemical analyses revealed virtually identical results in other visceral fat depots as in eWAT, including retroperitoneal WAT (rWAT) and mesenteric WAT (mWAT), with low temperature–dependent browning of these visceral fat depots (Supplemental Figure 3 and Supplemental Figure 4). In rWAT and mWAT, UCP1 expression was higher with –10°C and –10°C/–20°C than 4°C (Supplemental Figure 3 and Supplemental Figure 4). These findings generalize our conclusions that extremely low temperatures induce browning and UCP1-dependent thermogenesis of visceral fat.

Exposure of animals to –10°C and –10°C/–20°C markedly augmented UCP1-, mitochondrial prohibitin-, and endomucin-positive signals in s.c. WAT (Supplemental Figure 5, A and B). In particular, UCP1 protein expression was higher with –10°C/–20°C than 4°C (Supplemental Figure 5C). Thus, mitochondria-associated thermogenesis and adipose angiogenesis in s.c. fat became further elevated under extremely low temperature conditions. The key difference between visceral and s.c. WAT was that 4°C induced UCP1 expression and browning of s.c. fat but failed to switch on a browning phenotype in visceral fat (Figure 1 and Supplemental Figure 5). In striking contrast, UCP1 expression was nearly identical in interscapular BAT (iBAT) with 4°C, –10°C, and –10°C/–20°C, although levels were significantly higher than with 30°C (Supplemental Figure 6, A–C). Consistently, the prohibitin-positive mitochondrial content and endomucin-positive microvessel density in BAT was the same with –10°C and –10°C/–20°C as with 4°C (Supplemental Figure 6 A and B). Thus, activation of BAT peaked at 4°C, and further decreases of temperature could not augment UCP1 expression. In support of this notion, UCP1 protein levels in BAT with 4°C, –10°C, and –10°C/–20°C were indistinguishable (Supplemental Figure 6C).

### Genome-wide profiling of browning-related genes of low temperature–exposed visceral and s.c. WAT.

**Figure 2:**
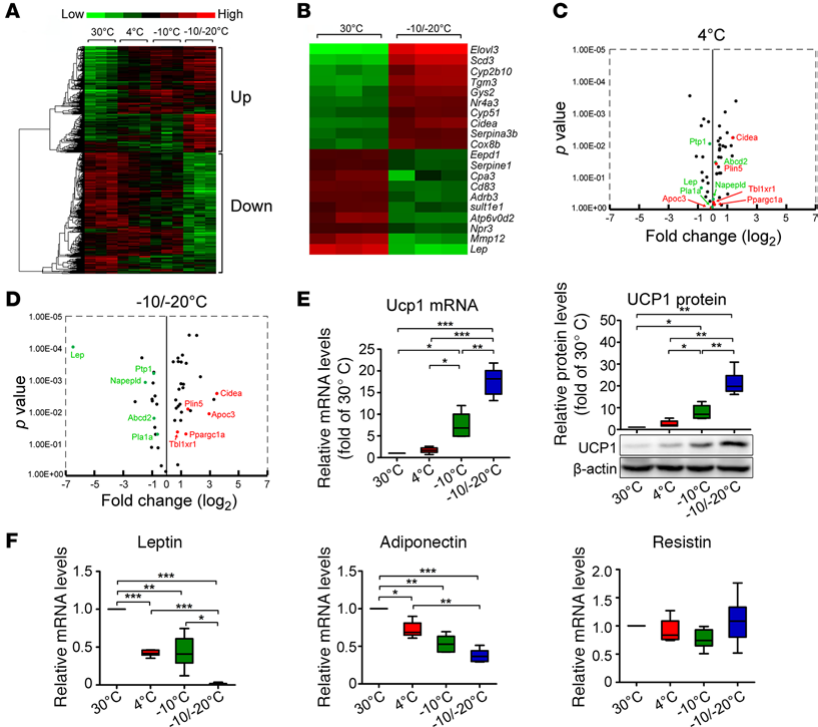
Genome-wide profiling, qPCR, and Western blot analyses of eWAT. (**A**–**D**) Genome-wide profiling of eWAT with exposure to various temperatures by heat map and volcano analyses (*n* = 3 samples per group). (**E**) Left panel: qPCR analysis of *Ucp1* expression in eWAT with exposure to various temperatures (*n* = 6 samples per group). Right panel: qPCR and Western blot analyses of UCP1 protein expression in eWAT (*n* = 6 samples per group). (**F**) Quantification of *Leptin*, *Adiponectin*, and *Resistin* mRNA levels in s.c. WAT exposed to various temperatures (*n* = 6 samples per group). *P < 0.05; **P < 0.01; ***P < 0.001, one-way ANOVA. Box-and-whisker plots show median (line within box), upper and lower quartile (bounds of box), and minimum and maximum values (bars).

To gain molecular signatures of browning of visceral WAT, we performed genome-wide profiling of gene expression in eWAT with 4°C, –10°C, and –10°C/–20°C. Heat map analysis of eWAT demonstrated that a cluster of genes was increasingly upregulated with decreasing temperature and another cluster was downregulated (Figure 2A). Further detailed analysis showed that elongation of very long chain fatty acids-like 3 (*Elovl3*), cell death–inducing DNA fragmentation factor, α subunit-like effector A (*Cidea*), and cytochrome c oxidase subunit 8b (*Cox8b*) were among the top 10 most upregulated genes with –10°C/–20°C (Figure 2B). Volcano plotting analyses further supported higher lipolysis in visceral fat with –10°C/–20°C (Figure 2, C and D, and Supplemental Tables 1 and 2).

*Ucp1* mRNA and protein expression became increasingly elevated with 4°C, –10°C, and –10°C/–20°C (Figure 2E), which provides further compelling evidence of activation of UCP1-dependent thermogenesis of visceral fat under extremely low-temperature conditions. Type II iodothyronine deiodinase (*Dio2*) has been implicated in cold-induced browning and lipolysis of WAT, as well as activation of BAT (26, 27). Similar to *Ucp1*, *Dio2* mRNA levels were increased with reducing temperature (Supplemental Figure 1D). Likewise, *Cidea* involved in cold-induced adipose metabolism was upregulated with reducing temperature (Supplemental Figure 1D). However, cytochrome c oxidase polypeptide 7A1 (*Cox7a1*) expression remained unchanged with all temperatures (Supplemental Figure 1D). The expression of peroxisome proliferator–activated receptor γ coactivator 1-α (*Pgc1α*), a transcriptional coactivator involved in energy metabolism, was modestly increased in eWAT with –10°C/–20°C relative to 30°C (Supplemental Figure 1E). Expression of *Prdm16*, a key factor causing browning of s.c. WAT at the posttranscriptional level (7), remained unchanged with all temperatures (Supplemental Figure 1E). *Ebf2* expression, involved in browning of WAT, remained unchanged with all cold temperatures (Supplemental Figure 1E).

In agreement with increased food intake, *leptin* levels were markedly reduced in eWAT with all cold groups (Figure 2F). With –10°C/–20°C, *leptin* level was barely detectable (Figure 2F). Consequently, circulating *leptin* levels successively decreased with reducing temperature (Supplemental Figure 1F). Additionally, levels of other factors, including *Adiponectin*, were lower in eWAT with –10°C/–20°C than 4°C, whereas *Resistin* levels remained unchanged in all groups (Figure 2F).

Although certain genes in cold-exposed s.c. WAT, including *Ucp1* and *leptin*, showed low temperature–dependent alterations as in eWAT, other transcription factors and adipokines were differentially expressed in visceral WAT and s.c. WAT under extreme low-temperature conditions (Figure 1, Supplemental Figure 1, and Supplemental Figure 7). In BAT, *Ucp1* mRNA expression peaked at 4°C, and lower temperature exposure did not further induce *Ucp1* expression (Supplemental Figure 8A). *Dio2* exhibited low temperature–dependent upregulation, but all other examined BAT activation–related factors — *Cidea, Prdm16,* and *Ebf2* (transcription factors)*; Cox7a1* (mitochondrial complex IV: cytochrome c oxidase subunit)*;* and *Pgc1α* (a transcriptional coactivator) *—* remained at similar levels with 4°C, –10°C, and –10°C/–20°C (Supplemental Figure 8, B and C). *Leptin* and *Adiponectin* levels were polarized during reducing environmental temperature (Supplemental Figure 8D). Again, extremely low –10°C/–20°C markedly downregulated *leptin* expression.

### Lower temperature–induced browning of visceral fat and body weight loss in obese mice fed a high-fat diet (HFD).

**Figure 3:**
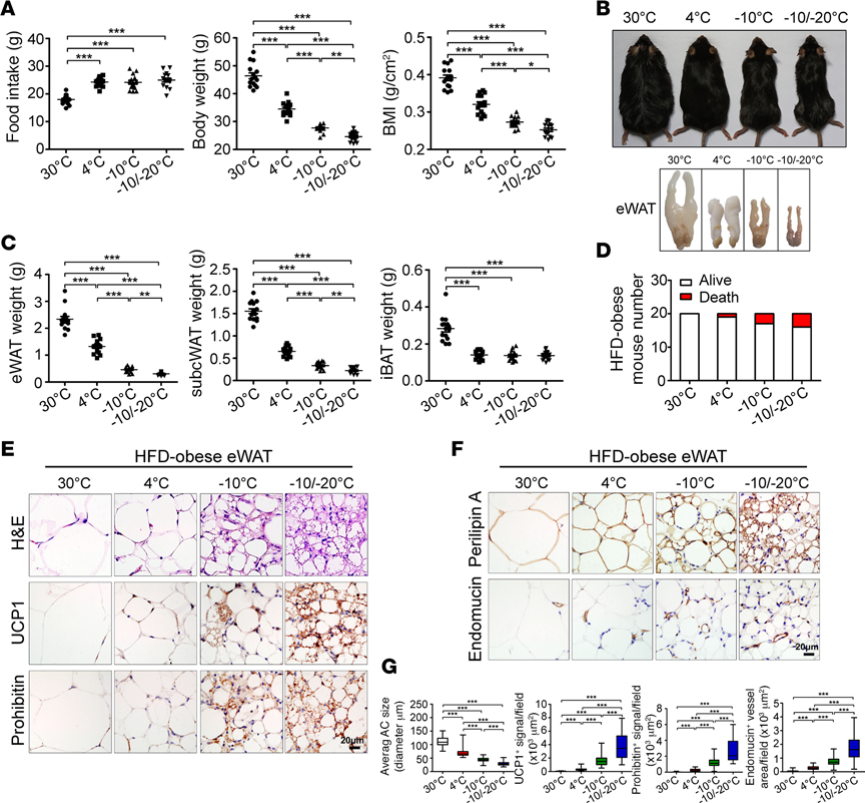
Food intake, body weight, BMI, and browning of visceral WAT of HFD-fed obese mice. (**A**) Food intake per week, body weight, and BMI of various temperature-exposed HFD-fed obese mice (*n* = 15 mice per group, data represent mean ± SEM, one-way ANOVA). (**B**) Mouse morphology and eWAT morphology. (**C**) Fat mass of eWAT, s.c. WAT, and iBAT of various HFD-fed obese mice (*n* = 15 mice per group, data represent mean ± SEM, one-way ANOVA). (**D**) Contingent survival and death of mice exposed to various temperatures (*n* = 20 mice per group). (**E** and **F**) H&E, UCP1, prohibitin, perilipin A, and endomucin staining of eWAT. Scale bars: 20 μm. (**G**) Quantification of AC size and UCP1-, prohibitin-, and endomucin-positive signals of eWAT (40 random fields from 8 mice in each group). *P < 0.05; **P < 0.01; ***P < 0.001, one-way ANOVA. Box-and-whisker plots show median (line within box), upper and lower quartile (bounds of box), and minimum and maximum values (bars).

For pathophysiological relevance, we designed similar experiments with HFD-fed obese animals. C57BL/6J mice were fed a 60% fat diet for 4 months before exposure to various cold conditions and continued feeding with the same HFD chow. Under all low-temperature conditions, food intake was significantly increased relative to 30°C (Figure 3A), with no difference in food intake between 4°C, –10°C, and –10°C/–20°C. Strikingly, obese mice with –10°C and –10°C/–20°C seemed considerably leaner than with 4°C and 30°C, and their body weight was successively reduced under reducing temperature (Figure 3, A and B). Total body weight was reduced about 40% after 4-week exposure to –10°C/–20°C relative to 30°C and about 20% relative to 4°C (Figure 3A). Accordingly, BMI was successively decreased in a similar low temperature–dependent manner as for body weight (Figure 3A). Remarkably, the eWAT weight with –10°C/–20°C was decreased more than 80 percent relative to 30°C and more than 50 percent relative to 4°C (Figure 3C). S.c. WAT mass also exhibited a similar low temperature–dependent reduction, although the degree of depot reduction was lesser than in eWAT (Figure 3C). In sharp contrast, iBAT mass remained unchanged in all low temperature–exposed animals (Figure 3C), indicating that activation of BAT peaked with 4°C cold exposure. Again, decreasing of low temperature resulted in an increased rate of mouse mortality (Figure 3D).

Histological examination of eWAT showed low temperature–dependent induction of UCP1 expression and mitochondrial content in HFD-fed obese animals (Figure 3E). UCP1 expression and mitochondrial content were markedly increased with –10°C/–20°C relative to 4°C (Figure 3, E and G). Similar to lean mice, in obese mice, adipocyte sizes were substantially reduced and microvessel density was increased in eWAT with –10°C and –10°C/–20°C (Figure 3, F and G). These findings were further validated in rWAT and mWAT (Supplemental Figure 9 and Supplemental Figure 10), which generalizes the conclusion that browning and UCP1 upregulation occurs in all visceral fat depots of obese animals under extreme cold conditions.

### Modest browning response of obese s.c. WAT to 4°C exposure.

With 4°C exposure, UCP-1 expression and mitochondrial content was only modestly increased in s.c. WAT in obese mice as compared with lean healthy mice (Supplemental Figure 11, A–D, and Supplemental Figure 5). In obese animals, these two important browning parameters together with microvessel density were increasingly elevated in s.c. WAT with –10°C and –10°C/–20°C (Supplemental Figure 11). Adipocyte sizes were reduced with reducing temperature (Supplemental Figure 11, A and B). With 4°C exposure, UCP1 level and mitochondrial content in s.c. WAT was lower in obese mice than lean in healthy mice (Supplemental Figure 11D and Supplemental Figure 5). Therefore, s.c. WAT was less susceptible to cold-induced browning in obese than lean mice. In contrast to s.c. WAT, in iBAT, –10°C and –10°C/–20°C did not further increase mitochondrial content, UCP1 expression, and microvessel density relative to 4°C in obese mice (Supplemental Figure 12), which suggests that activation of BAT peaked under 4°C.

Consistent with increased UCP1 protein level, *Ucp1* mRNA level was markedly increased by more than 10-fold in s.c. WAT with –10°C and –10°C/–20°C relative to 4°C (Supplemental Figure 13A). Similarly, levels of browning-related transcription factors including *Dio2*, *Cidea*, *Cox7a1*, and *Pgc1α* were increasing elevated in a low temperature–dependent manner (Supplemental Figure 13, B and C). Surprisingly, level of *Prdm16*, a transcription factor involved in browning of s.c. WAT, was decreased with all cold temperatures (Supplemental Figure 13C). Furthermore, *leptin* level was almost undetectable with –10°C/–20°C (Supplemental Figure 13D).

### Improvement of insulin sensitivity and blood lipid profiles.

**Figure 4:**
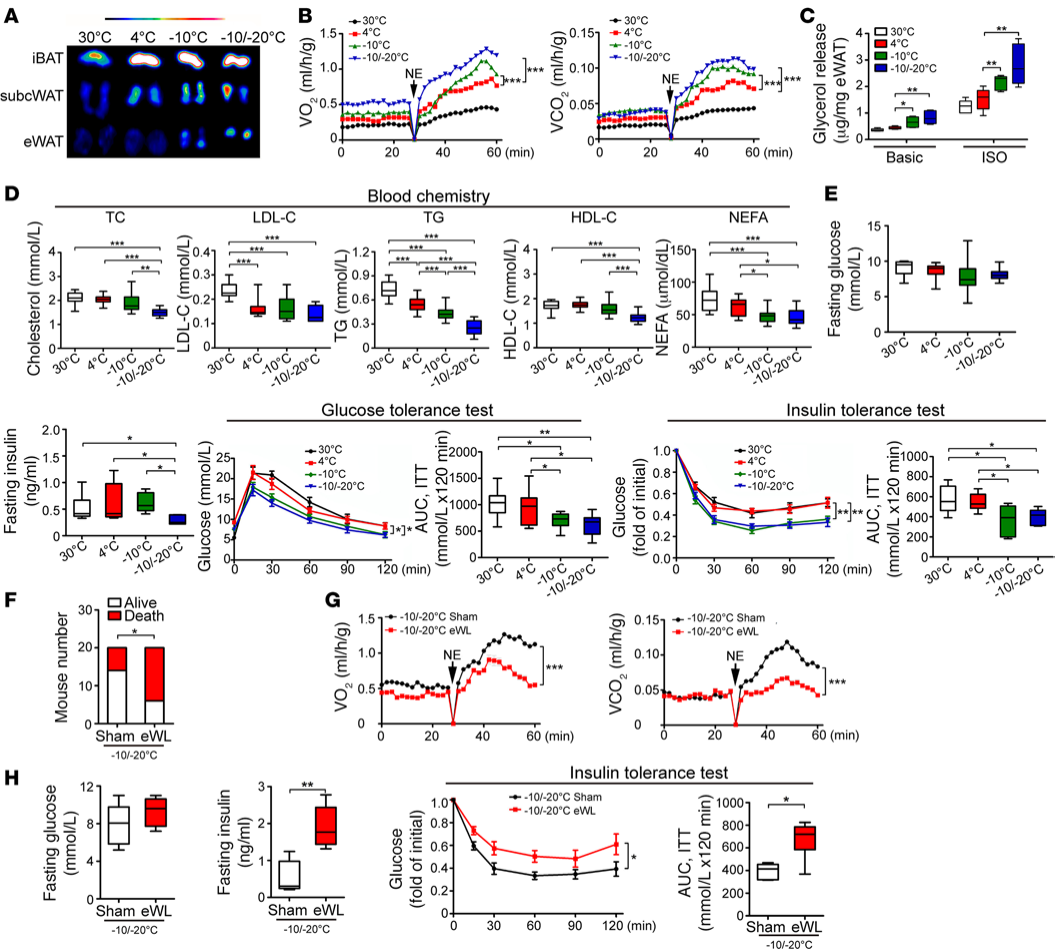
Micro-PET imaging, nonshivering thermogenesis, lipolysis, blood lipid profiling, and glucose metabolism. (**A**) Micro-PET imaging of iBAT, s.c. WAT, and eWAT. (**B**) Metabolic rates of O_2_ consumption and CO_2_ production in response to norepinephrine (NE) (*n* = 5 mice per group, two-way ANOVA, data represent mean ± SEM). (**C**) Glycerol release from eWAT of various groups (*n* = 6 samples per group). One-way ANOVA. (**D**) Blood lipid profile of cholesterol, low-density lipoprotein cholesterol (LDL-C), triglycerides (TG), high-density lipoprotein cholesterol (HDL-C), and nonesterified fatty acids (NEFAs) (*n* = 15 samples per group). One-way ANOVA. (**E**) Fasting glucose, fasting insulin, insulin-tolerance test, and glucose-tolerance test with exposure to various temperatures (*n* = 8–10 animals per group). AUC of insulin-tolerance test and glucose-tolerance test. One-way ANOVA. (**F**) Contingency survival and death of eWAT-less (eWL) and sham-operated (Sham) mice exposed to –10°C/–20°C (*n* = 20 animals per group, χ^2^ test). (**G**) Metabolic rates of O_2_ consumption and CO_2_ production of eWL and sham-operated mice in response to norepinephrine (NE) under –10°C/–20°C (*n* = 5 animals per group). Two-way ANOVA, data represent mean ± SEM. (**H**) Fasting glucose, fasting insulin, and insulin-tolerance test of eWL and sham-operated mice exposed to –10°C/–20°C (*n* = 6 animals per group). AUC of insulin-tolerance test. *P < 0.05; **P < 0.01; ***P < 0.001, 2-tailed *t* test for fasting glucose, fasting insulin, and AUC; two-way ANOVA for insulin-tolerance test. Data represent mean ± SEM. Box-and-whisker plots show median (line within box), upper and lower quartile (bounds of box), and minimum and maximum values (bars).

We used positron emission tomography analysis with fludeoxyglucose F 18 (^18^F-FDG) as a glucose substitute to obtain direct evidence of browning of visceral WAT. ^18^F-FDG uptake in eWAT was enhanced with –10°C and –10°C/–20°C as compared with 4°C (Figure 4A). Similarly, ^18^F-FDG uptake in s.c. WAT was increasingly enhanced with reducing temperature (Figure 4A). However, ^18^F-FDG uptake in iBAT was not altered under all cold conditions, which supports that further reducing temperature did not elevate its metabolic capacity. Interestingly, norepinephrine-stimulated nonshivering thermogenesis (NST), as measured by O_2_ consumption and CO_2_ production, was markedly increased with –10°C and –10°C/–20°C relative to 4°C (Figure 4B). Also, increasing lipolysis was detected in eWAT with reducing temperature (Figure 4C). Blood lipid analysis supported a super-active lipolysis with –10°C and –10°C/–20°C, for reducing blood levels of triglycerides (TG), and nonesterified fatty acids (NEFAs) (Figure 4D). Additionally, blood cholesterol (TC) and high-density lipoprotein cholesterol (HDL-C) levels were markedly decreased with reducing temperature, although low-density lipoprotein cholesterol (LDL-C) levels remained similar in all cold-exposed groups (Figure 4D).

The fasting glucose levels of healthy mice were not altered in all experimental conditions (Figure 4E). However, insulin tolerance was improved with –10°C and –10°C/–20°C relative to 4°C (Figure 4E). Also, fasting insulin level was lower with –10°C/–20°C than other temperatures (Figure 4E). With –10°C and –10°C/–20°C, glucose tolerance was significantly improved relative to 4°C (Figure 4E). Thus, exposing mice to –10°C- and –10°C/–20°C significantly improved insulin sensitivity in healthy mice.

### Low temperature–induced browning of visceral fat contributes to global NST and insulin sensitivity.

To provide further evidence of visceral fat contributing to altered global metabolism under extremely cold conditions, we surgically removed eWAT from mice. After adaptation in 18°C, 4°C, and –10°C environments, mice were exposed to –10°C/–20°C. Removal of eWAT resulted in the death of most animals under this temperature (Figure 4F and Supplemental Figure 2B). For the remaining animals, NST was significantly impaired as compared with sham-operated mice (Figure 4G). Therefore, browning of eWAT significantly participated in global metabolism. Interestingly, eWAT removal markedly increased insulin resistance by increasing fasting insulin level (Figure 4H). In support of this notion, insulin sensitivity was significantly decreased in eWAT-less (Figure 4H). Altogether, these findings demonstrate that visceral fat significantly contributes to regulation of global metabolism and insulin sensitivity under extremely cold conditions.

### Knockdown of UCP1 in eWAT attenuates global NST and insulin sensitivity.

**Figure 5:**
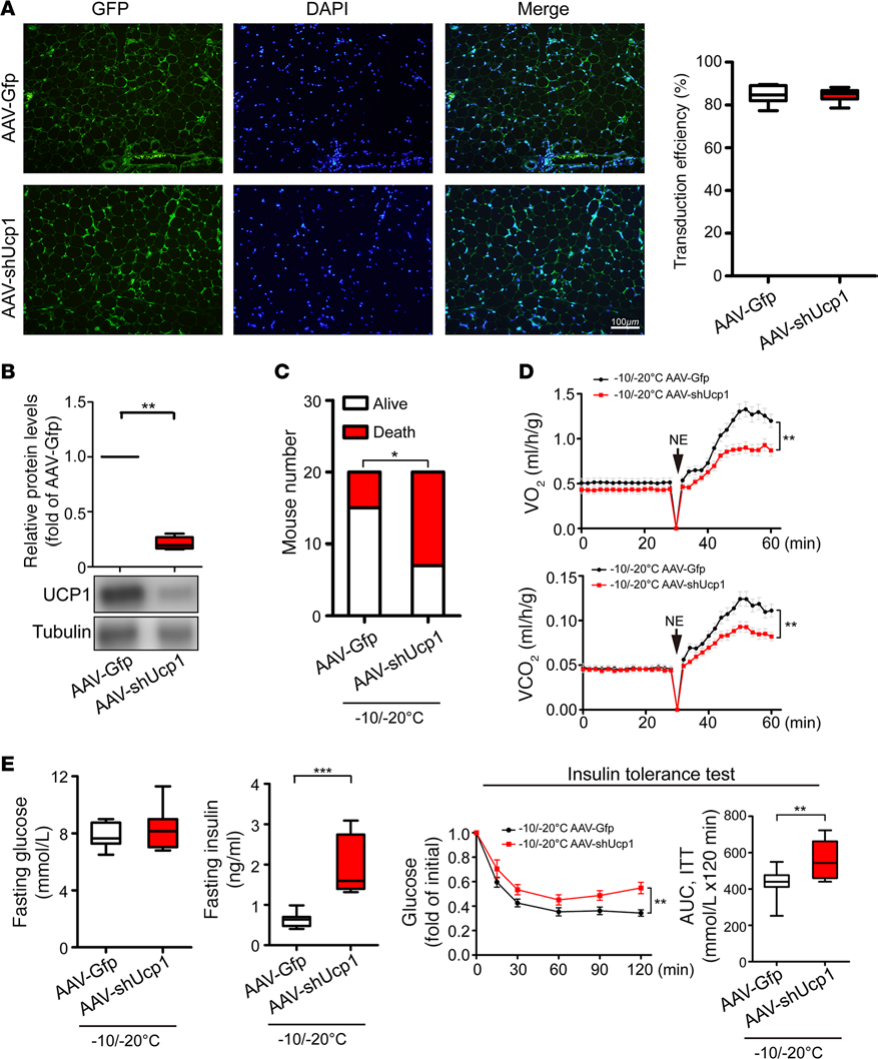
Impacts of UCP1 knockdown on global metabolic functions under an extreme cold condition. (**A**) Immunohistological analysis of AAV transduction efficiency. AAV-Gfp– and AAV-shUcp1–transduced eWAT expressed GFP (green). The sections were counter-stained with DAPI (blue). Transduction efficiencies were quantified (*n* = 4 samples per group, 2-tailed *t* test). (**B**) Knockdown efficiency of endogenous UCP1 protein was analyzed by Western blot analysis (*n* = 6 samples per group, 2-tailed *t* test). (**C**) Contingent survival and death of AAV-Gfp– and AAV-shUcp1–transduced mice under –10°C/–20°C (*n* = 20 mice per group, χ^2^ test). (**D**) Metabolic rates of O_2_ consumption and CO_2_ production of AAV-Gfp– and AAV-shUcp1–transduced mice in response to norepinephrine (NE) under –10°C/–20°C (*n* = 5 mice per group). Two-way ANOVA, data represent mean ± SEM. (**E**) Fasting glucose, fasting insulin, and insulin-tolerance test of AAV-Gfp– and AAV-shUcp1–transduced mice under –10°C/–20°C (*n* = 10 mice per group). AUC of insulin-tolerance test. *P < 0.05; **P < 0.01; ***P < 0.001. 2-tailed *t* test for fasting glucose, fasting insulin, and AUC; two-way ANOVA for insulin-tolerance test. Data represent mean ± SEM. Box-and-whisker plots show median (line within box), upper and lower quartile (bounds of box), and minimum and maximum values (bars).

Since removal of visceral fat might impair its substrate supply for thermogenesis in s.c. browning WAT and BAT, attenuation of global metabolism and insulin sensitivity might not directly relate to eWAT-triggered thermogenesis. To exclude this possibility, we took a loss-of-function approach by knocking down UCP1 in eWAT, leaving the eWAT depot intact in experimental animals. To achieve this goal, a bicistronic adeno-associated vector expressing shUcp1 (AAV-shUcp1) and enhanced GFP was constructed and delivered to eWAT. Histological analysis showed that more than 80% of adipocytes of AAV-shUcp1–transduced eWAT displayed GFP positivity (Figure 5A), indicating high efficiency of transduction. Consistent with the high efficiency of transduction, analysis of UCP1 protein expression showed that AAV-shUcp1 effectively blocked UCP1 protein expression under the –10°C/–20°C extreme cold condition (Figure 5B). Exposure of AAV-Ucp1–transduced animals to –10°C/–20°C markedly increased the death rate relative to the control AAV-GFP–transfected mice (Figure 5C). Similar to eWAT-removal experimental settings, AAV-shUcp1–transduced eWAT showed impaired global metabolism (Figure 5D). AAV-shUcp1–transduced animals exhibited increased insulin resistance (Figure 5E). These findings validate the fact that UCP1-dependent NST in visceral fat under extreme cold conditions is beneficial for metabolism, insulin sensitivity, and animal survival.

### Improvement of insulin sensitivity and hepatic steatosis in obese mice.

**Figure 6:**
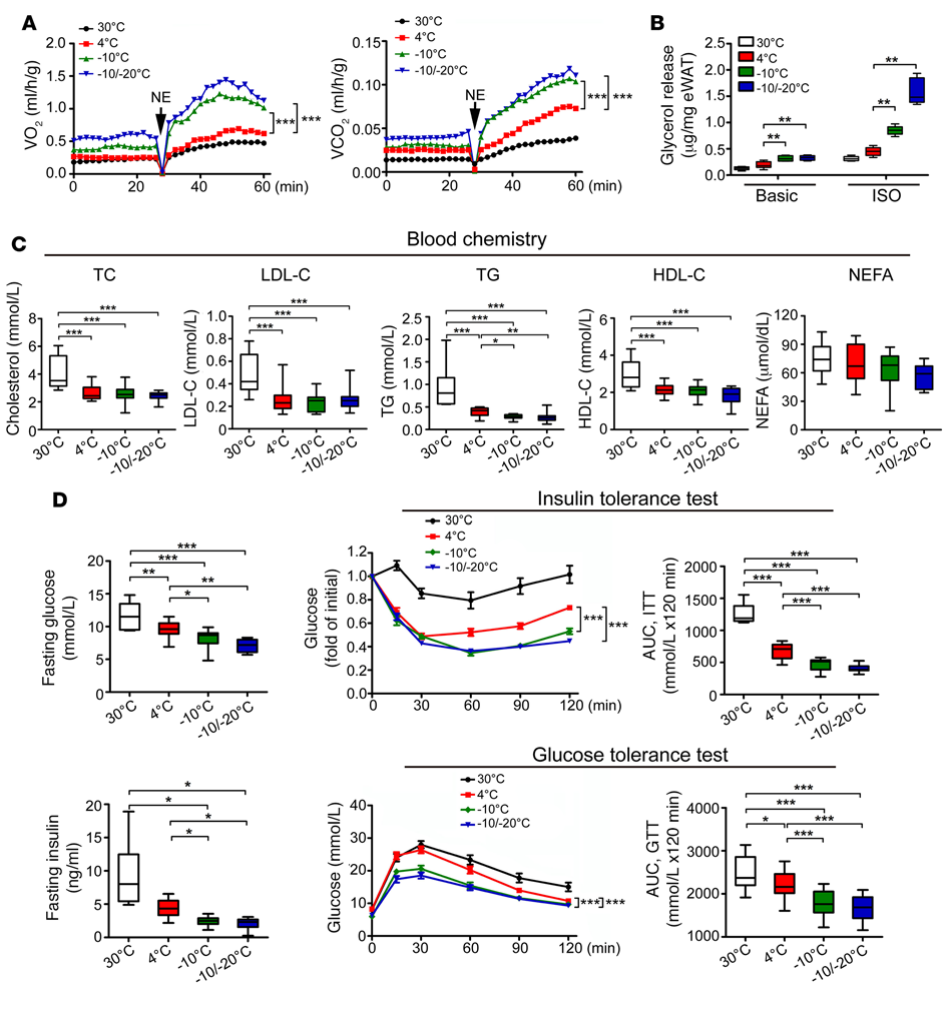
Nonshivering thermogenesis, lipolysis, blood lipid profiling, and glucose metabolism in HFD-fed obese mice. (**A**) Metabolic rates of O_2_ consumption and CO_2_ production of HFD-induced obese mice in response to NE (*n* = 5 mice per group). Two-way ANOVA. Data represent mean ± SEM. (**B**) Glycerol release from eWAT of various HFD-induced obese mice (*n* = 6 samples per group). One-way ANOVA. (**C**) Blood lipid profile of cholestero (TC), LDL-C, TG, HDL-C, and NEFAs of HFD-induced obese animals (*n* = 15 samples per group). One-way ANOVA. (**D**) Fasting glucose, fasting insulin, insulin-tolerance test, and glucose-tolerance test of HFD-induced obese mice exposed to various temperatures (*n* = 8 samples per group for 30°C; *n* = 11–15 samples per group for other groups). AUC of insulin-tolerance test and glucose-tolerance test. *P < 0.05; **P < 0.01; ***P < 0.001. One-way ANOVA for fasting glucose, fasting insulin, and AUC; two-way ANOVA for insulin-tolerance test and glucose-tolerance test. Data represent mean ± SEM. Box-and-whisker plots show median (line within box), upper and lower quartile (bounds of box), and minimum and maximum values (bars).

Similar to healthy lean mice, in obese animals, –10°C and –10°C/–20°C increased the metabolic rate as compared with 4°C (Figure 6A). Along with increased metabolic rate under extremely low temperature, lipolysis was increased (Figure 6B). Levels of blood lipids, including TG, TC, LDL-C, and HDL-C, were improved under cold conditions, although with no differences between 4°C, –10°C, and –10°C/–20°C (Figure 6C). Notably, fasting glucose level was decreased with 4°C, –10°C, and –10°C/–20°C relative to thermoneutrality (Figure 6D). Insulin sensitivity was improved with –10°C and –10°C/–20°C relative to 30°C and 4°C (Figure 6D). Fasting insulin level was lower with –10°C and –10°C/–20°C than 30°C and 4°C (Figure 6D). Similarly, –10°C and –10°C/–20°C significantly improved glucose tolerance (Figure 6D).

**Figure 7:**
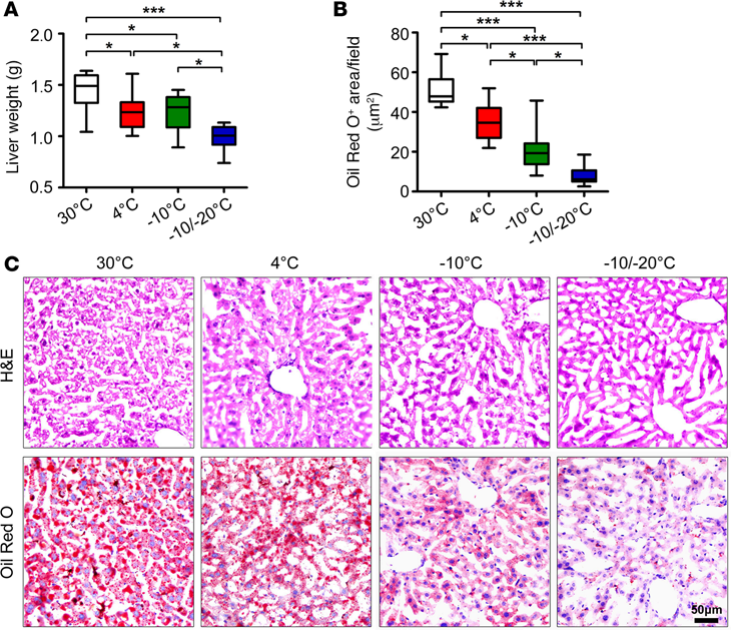
Liver steatosis in HFD-fed obese mice. (**A**–**C**) Weight, H&E staining, and Oil Red O staining of liver tissues from HFD-induced obese mice exposed to various temperatures (*n* = 8 animals per group). Scale bar: 50 μm. Oil Red O–positive signals were quantified from 20 random fields. *P < 0.05; ***P < 0.001. One-way ANOVA.

Because obese animals often develop steatosis in the liver, we examined liver tissues under different temperature conditions. Liver weight was reduced with –10°C/–20°C relative to 4°C, although 4°C also reduced liver weight as compared with 30°C (Figure 7A). Lipid droplet content was markedly reduced with –10°C and–10°C/–20°C, with a more than 5-fold reduction with –10°C/–20°C relative to 4°C, as measured by Oil Red O staining (Figure 7, B and C). Therefore, exposing obese mice to very low temperatures markedly improved insulin sensitivity and liver steatosis.

HFD-fed obese eWAT showed increasingly upregulated *Ucp1*, *Dio2*, *Cidea*, *Cox7a1*, and *Pgc1α* with reducing temperature (Supplemental Figure 14, A–C). However, levels of *Prdm16*, *Ebf2*, *Leptin*, and *Adiponectin* expression levels were decreasingly downregulated (Supplemental Figure 14, C and D).

## Discussion

**Figure 8:**
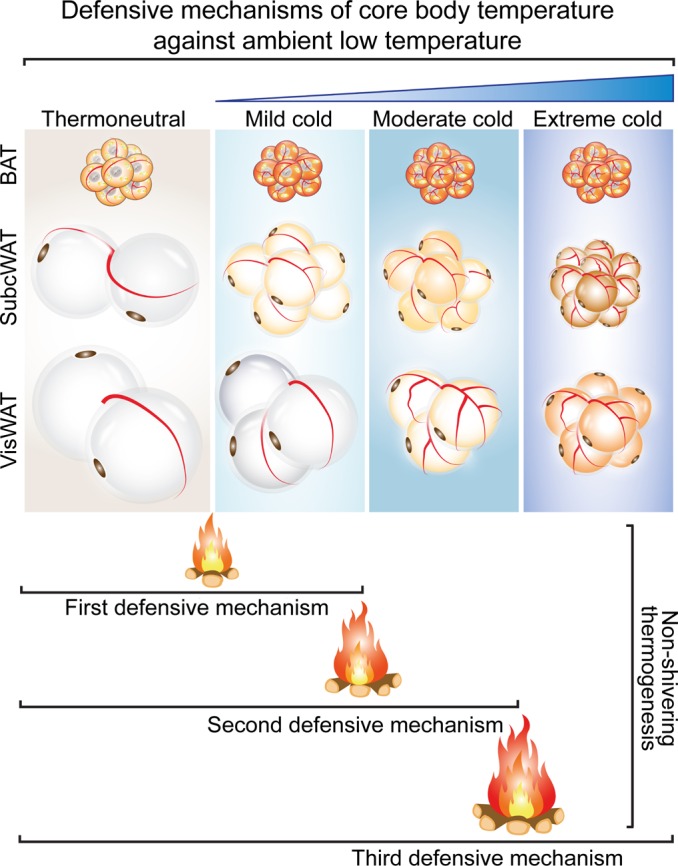
Schematic diagram of successive activation of defensive mechanisms against decreasing environmental cold. Under mild cold such as 4°C exposure, activation of the BAT-nonshivering thermogenesis and modest browning of s.c. WAT are sufficient to maintain core body temperature. However, visceral WAT remains thermogenically inactive. Further decreased environmental temperature — to –10°C, for example — enhances browning of s.c. WAT and triggers modest browning of visceral fat to generate nonshivering heat. With extreme cold such as –10°C/–20°C, browning of visceral fat markedly contributes to nonshivering thermogenesis to main core body temperature. Additionally, browning of visceral fat increases insulin sensitivity and improves liver steatosis in obese mice.

Here, we uncovered a mechanism that underlies low temperature–dependent activation of NST in visceral fat. From our compelling data from experimental mice, we propose three successive mechanisms of maintaining core body temperature against decreasing environmental temperature (Figure 8). With exposure to mild cold, such as 4°C, full activation of BAT and modest browning of s.c. WAT are sufficient for rodents to maintain core body temperature via NST. This is the first-line defensive machinery that involves generating nonshivering heat from BAT and s.c. adipose depots. However, this BAT-committed NST is not sufficient for further decreased environmental temperature. In the second-line defense, BAT-produced thermogenesis peaks, and increasing browning of s.c. WAT offers a compensative mechanism to sustain core body temperature. Under an extremely cold condition, such as –20°C, maximal activation of BAT and browning of s.c. WAT produces only limited nonshivering heat, which no longer sustains the critical core body temperature needed for survival. The remaining reserve energy stored in visceral fat starts to be burned as the third-line defense for heat production. Even though the environmental temperature remains extremely low, mice are able to sustain an adequate core body temperature for survival. At the molecular level, our findings suggest that browning of s.c. WAT and visceral WAT may involve different regulatory mechanisms, although sympathetic activation is common for both. For example, in both lean and obese animals, PRDM16, an essential transcription factor for browning s.c. WAT, is constantly downregulated in extremely low temperature–exposed visceral fat. This notion agrees with a recent published work showing that PRDM16 is expressed at a very low level in visceral WAT as compared with s.c. WAT (*7*). Thus, browning of visceral fat may involve a PRDM16-independent mechanism.

The fact that cold acclimation switches s.c. WAT to a brownish (beige/brite) phenotype for NST has been known in rodents for decades (28). Visceral fat is considered anatomically and functionally different from s.c. fat and remains indolent on cold exposure. This conclusion was exclusively based on the constant exposure of experimental mice to 4°C. However, such a constant temperature-equipped environment does not exist in a given natural environment. Under physiological conditions, mammals, including humans, in a natural environment are relentlessly exposed to temperature variations; especially in a large part of the globe, extremely low temperature environments exist during cold seasons. Thus, our findings are physiologically relevant. Perhaps, even hibernating animals use this three-line defense mechanism of successively elevating NST of BAT, s.c. WAT, and visceral WAT to protect against extremely cold weather. This possibility warrants further investigation.

In a recent study, Spiegelman and colleagues showed that browning of s.c. WAT substantially contributed to 4°C cold-altered global metabolism, insulin sensitivity, and liver steatosis (*7*). Here, we show similar findings with visceral fat under extremely cold conditions. Surgical removal of eWAT resulted in lethality of most animals, which suggests that mice could no longer sufficiently maintain their core body temperature. For the remaining animals, lack of eWAT had a significant effect on global metabolism, with significantly impaired thermogenesis. To our knowledge, our work demonstrates for the first time that browning of visceral WAT contributes to global metabolic changes under a physiologically tolerable cold condition. Consequently, global impairment of thermogenesis of visceral fat decreases insulin sensitivity. This claim sounds paradoxical because visceral fat is considered “bad fat” and contributes to insulin resistance, type 2 diabetes, and cardiovascular diseases. However, this conclusion is based on the association of visceral fat obesity with disease development under thermoneutrality conditions. We show that, under extremely cold conditions, visceral fat can have a positive effect for body weight reduction, insulin sensitivity, and even survival. Without visceral fat, animals would have difficulties maintaining core body temperature, basic metabolism, and survival. Similarly, knockdown of UCP1 in visceral fat also produced functional impacts on global metabolism impairment and insulin resistance. Thus, our views on visceral fat modulating physiological and pathological functions should be altered according to the environmental context. Our view provides a new concept of turning “harmful” or “bad” visceral fat into “beneficial” or “good” fat for fighting against obesity and metabolic diseases.

We also show that extremely low temperature–induced browning of visceral fat also occurs in HFD-induced obese mice. Even though obese mice show increased food intake during cold exposure, the total body weight, especially the eWAT weight, was markedly decreased. Of note, obese mice showed improved glucose clearance, insulin sensitivity, and liver steatosis under extremely cold conditions. If these findings can be proven in human obese subjects, exposure of obese-associated diabetic patients to very low temperature would offer a novel noninvasive therapy for treatment of obesity and diabetes. The principle of a cold-based therapeutic approach is based on converting harmful visceral fat to benefit glucose uptake and excessive energy consumption. A treatment based on burning obese visceral fat might have implications for obesity and diabetes. Of course, caution should be paid to obese patients who have other complications, such as atherosclerosis-related cardiovascular disease, because cold exposure–triggered lipolysis might increase LDL-C remnants in the circulation (29). Taken together, our present work proposes switching harmful visceral fat to an energy-burning machinery that may be beneficial for treating obese and diabetic patients.

## Methods

Extended Experimental Procedures are included in the Supplemental Information.

### Animals.

Male 7- to 8-week-old C57BL/6J mice were purchased from the Beijing HFK Bioscience Co. Mice were kept at 22°C before random grouping. For obese animal experiments, C57BL/6J male mice were fed a 60% lipid composition HFD for 4 months before cold acclimation.

### Cold acclimation.

The cold facilities were designed for a 12-hour light and 12- hour dark rhythm, and mice were caged 5 per cage. Cold chambers of 4°C, –10°C, and –10°C/–20°C were equipped with several thermometers to monitor the accuracy of inner temperature. Before cold exposure, all mice were adapted at 18°C for 1 week and then transferred to 4°C for another week. One group remained at 4°C for the entire experimental duration. Other groups of mice were kept at –5°C during daytime (12 hours) and at 4°C during nighttime (12 hours) for 3 consecutive days. Mice were then exposed to –10°C for 12 hours during daytime and to 4°C for 12 hours during nighttime for the rest of the week. One group was further maintained at –10°C for 24 hours for 3 weeks. The –10°C/–20°C group was transferred to –20°C for 12-hour daytime and –10°C for 12 h nighttime during the next 3 weeks. For –10°C- and –10°C/–20°C-exposed animals, fresh drinking water was frequently changed. Extra snow and frost was put in the cage floor to make sure mice had free access to moisture. For thermoneutrality, mice were directly transferred to 30°C and maintained at this temperature for the duration equivalent to cold exposure. Food intake per week was measured by subtracting the remaining food from the total amount of food. Animal physical activities were monitored by a video camera, and 1-hour record was randomly chosen for analysis. Out-nest activities were calculated for each group.

### Antibodies and HFD.

Antibodies included rabbit anti-UCP1 (ab10983, Abcam), rabbit anti-prohibitin (ab28172, Abcam), rabbit anti–perilipin A (ab3526, Abcam), rat anti–mouse endomucin (14-5851, eBioscience) and mouse anti–β-actin (TA346894, ZSGB-Bio). The HRP DAB detection system for rabbit or rat antibodies (ZLI-9018, ZSGB-Bio) was used for immunohistochemical staining. Rodent HFD with 60% kcal% fat was from Research Diets.

### Tissue sample collection.

For measuring blood lipid levels and fasting insulin, mice were starved for 6–8 hours. Blood samples were collected by retro-orbital puncturing or through the cardiac apex of isoflurane- or pentobarbital-anesthetized animals. After sacrifice, various adipose depots and livers were quickly dissected and weighed. Some tissues were kept in liquid nitrogen and stored at –80°C until further extraction of RNA and protein. A fraction of tissues was fixed in 4% paraformaldehyde (PFA) for future histological analysis.

### Histology and IHC.

H&E staining was performed according to our previously published methods (23, 29–31). Various adipose depots were collected, fixed with 4% PFA, and embedded in paraffin. Tissue samples were cut in 5-μm–thick slides and used for immunohistochemical staining. After dewaxing and antigen retrieval with a citrate buffer (pH 6.0), followed by treatment with 3% H_2_O_2_, tissue slides were blocked with 5% goat nonimmune serum (CW0130S, CWBiotech) for 30 minutes at 37°C. Primary antibodies including UCP1, prohibitin, perilipin A, and endomucin were incubated at 4°C overnight. HRP-conjugated secondary antibodies were added the next day. A DAB kit (ZSGB-Bio) was used for color development. Liver cryosection slides were stained with 0.5% Oil Red O (Sigma–Aldrich) for 10 minutes at room temperature, then washed with water at 37°C for a few seconds. The nuclei were counterstained with hematoxylin.

### Gene microarray analysis.

Total RNA was extracted from 30°C-, 4°C-, –10°C-, and –10°C/–20°C-exposed eWAT in triplicates. Affymetrix Gene Chip Mouse Exon 1.0 ST Array was used. The Gene Expression Omnibus (GEO) accession number for the dataset reported in this paper is GSE74062.

### Real-time PCR.

Total RNA was extracted from adipose tissues using TRIzol Reagent (Invitrogen) according to the manufacturer’s instruction. cDNA was synthesized using a PrimeScript RT reagent Kit (Takara). Quantitative PCR (qPCR) involved use of UltraSYBR Mixture (CWBiotech) with a Bio-Rad iQ5 system. The 2^-ΔΔCT^method was used to assess relative mRNA expression levels. qPCR primers were in Supplemental Table 3.

### Western blot analysis.

Total proteins from adipose tissues were separated by 10% SDS-PAGE and transferred to a PVDF membrane, which was blocked with 5% nonfat milk and incubated with primary antibodies at 4°C overnight. Transferred blots were developed with a chemiluminescent reagent (Millipore).

### Micro-PET imaging.

Prior to injection, mice were fasted for about 3 hours and were i.v. injected with 200–300 μCi ^18^F-FDG. After injection, the 30°C group was kept at 30°C, while the 4°C, –10°C, and –10°C/–20°C groups were kept in cold. After 1 hour, adipose depots were quickly dissected and scanned by the Inveon Dedicated PET System (Siemens).

### Core body temperature.

Core body temperature of various treated and nontreated animals was measured via a rectal temperature probe thermometer (Winstrument) between 10–12 am.

### Indirect calorimetry.

After 4-week exposure to different temperatures, NST capacity was measured by an open-circuit system (Sable) as described (23, 29, 32). Animals were anesthetized with pentobarbital (90 mg/kg, i.p.), and O_2_ consumption as well as CO_2_ release were measured for 30 minutes at 33°C. After measuring the basal metabolic rate, mice were injected with norepinephrine (1 mg/kg, s.c.). O_2_ consumption and CO_2_ release were recorded for the following 90 minutes.

### Measurement of adipose glycerol.

To assess lipolysis, eWAT was surgically dissected and washed several times with PBS. Tissue pieces (~50 mg) were incubated at 37°C with DMEM (Gibco) containing 2% BSA (Wako Chemicals) with or without 10 mM isoproterenol (Sigma-Aldrich) for 4 hours. The glycerol content was measured in the culture medium using commercial kits (Sigma-Aldrich).

### Blood lipid and leptin analysis.

Serum levels of TC, LDL-C, TG, HDL-C, and NEFA were measured using commercial kits (Roche Diagnostics). Serum leptin levels were measured by an ELISA (Abcam) assay.

### Blood glucose, blood insulin, insulin-tolerance test, and glucose-tolerance test.

Blood glucose strips and the Accu-Chek glucose meter (Roche Diagnostics) were used to measure fasting glucose and to monitor blood glucose levels during insulin and glucose-tolerance tests (33). Fasting serum insulin was measured using commercial ELISA kits from Mercodia. Prior to insulin-tolerance test, all mice fasted for 6 hours and then were i.p. injected with 0.5 U human insulin/kg body weight (lean healthy mice) or 0.75 U human insulin/kg body weight (obese mice). Blood glucose levels were determined before injection and at 15, 30, 60, 90, and 120 minutes after the injection, as described above. For the glucose-tolerance test, all mice fasted for 12 hours and were i.p. injected with 2 g glucose/kg body weight. Blood glucose levels were measured before and at 15, 30, 60, 90, and 120 minutes after the injection.

### Surgical removal of eWAT.

Prior to 18°C adaptation, both lateral eWAT depots were surgically removed as described (34). Briefly, eWAT was removed by blunt dissection through a vertical midline incision. eWAT fat pads were removed without compromising blood supply to the testes. For the sham operation, the abdominal cavity was incised, and eWAT was mobilized but not excised.

### UCP1 knockdown by an AAV delivery approach.

AAV-Gfp and AAV-shUcp1 were designed and purchased from the Shanghai Genechem Co. Ltd. The titers of AAV-Gfp and AAV-shUcp1 is approximately 5 × 10^12^ virus genomes/ml. Prior to 18°C adaptation, the abdominal cavity was surgically exposed and both lateral eWAT fat pads of each mouse were injected with 10 μl AAV-Gfp or AAV-shUcp1 at multiple sites. The injected mice were kept at 18°C for 2 weeks and subsequently transferred to 4°C and –10°C/–20°C as described above.

### Statistics.

All data were presented as the mean ± SEM. Data analysis involved unpaired 2-tailled Student’s *t* test for two groups and one-way ANOVA for more than two groups. Data for insulin and glucose-tolerance tests were analyzed by two-way ANOVA. *P* < 0.05 was considered statistically significant.

### Study approval.

All animal studies complied with the Management Rules of the Chinese Ministry of Health and were reviewed and approved by the Local Ethical Committee of Qilu Hospital, Shandong University, Jinan, Shandong Province, China.

## Author contributions

YC generated ideas of this study, analyzed the results, and wrote the manuscript. XY, WS, and MZ performed most experiments and analyzed the data and the results. XY and WS helped to write the manuscript. MD performed many experiments and analyzed the data. YZ actively participated in discussions and supervised XY, MZ (Department of Microbiology), and MD for performing the experiments. YC and XY designed all experiments and all figures. SL, TS, ZG, CF, YG, YW, HL, CZ, JY, CC, YS, MZ, XZ, MS, RZ, and NJS contributed to discussion, experimental designs, and performing actual experiments.

## Supplementary Material

Supplemental data

Supplemental Table 1

Supplemental Table 2
