# Supplementary material for: Switching harmful visceral fat to beneficial energy combustion improves metabolic dysfunctions
Source: JCI Insight. 2017 Feb 23;2(4):e89044. doi: 10.1172/jci.insight.89044 (PMC5313060; doi:10.1172/jci.insight.89044)
Supplement: Supplemental data [file jciinsight-2-89044-s001.pdf]

Supplementary Information

Switching harmful visceral fat to beneficial energy combustion improves metabolic dysfunctions

Xiaoyan Yang[1,2,3,#], Wenhai Sui[1,2,#], Meng Zhang[1,2], Mei Dong[1,2], Sharon Lim[2], Takahiro Seki[2], Ziheng Guo[4], Carina Fischer[2], Huixia Lu[1], Cheng Zhang[1], Jianmin Yang[1], Meng Zhang[1], Yangang Wang[5], Caixia Cao[5], Yanyan Gao[5], Xingguo Zhao[6], Meili Sun[7], Yuping Sun[7], Rujie Zhuang[8], Nilesh J Samani[9], Yun Zhang[1*], and Yihai Cao[1,2,9*]

[1]The Key Laboratory of Cardiovascular Remodeling and Function Research, Chinese Ministry of Education and Chinese Ministry of Public Health, Shandong University Qilu Hospital, Jinan, Shandong 250012, China

[2]Department of Microbiology, Tumor and Cell Biology, Karolinska Institutet, S-171 77, Stockholm, Sweden

[3]Department of Cardiology, Beijing Chaoyang Hospital, Capital Medical University, Beijing 100020, China

[4]West China School of Medicine, Sichuan University, Chengdu 610041, China

[5]Department of Endocrinology and Metabolism, Affiliated Hospital of Qingdao University, Qingdao, 19 266003, China

[6]Department of Otolaryngology, Shandong University Qilu Hospital, Jinan, Shandong 250012, China

[7]Department of Oncology, Jinan Central Hospital, Shandong University, Jinan, Shandong, China.

[8]The TCM Hospital of Zhejiang Province, Hangzhou 310006, Zhejiang, China

[9]Department of Cardiovascular Sciences, University of Leicester and NIHR Leicester Cardiovascular Biomedical Research Unit, Glenfield Hospital, Leicester, LE3 9QP, UK.

Key words: Visceral fat, white fat browning, non-shivering thermogenesis, adipose metabolism, obese

Running title: Freezing temperature induces visceral fat browning

[#]These authors contributed equally to this work.
*Correspondence, galley proofs and reprint requests should be primarily addressed to: Yihai Cao, M.D., Ph.D., Department of Microbiology, Tumor and Cell Biology, Karolinska Institutet, 171 77 Stockholm, Sweden. Tel: (+46)-8-5248 7596, Fax: (+46)-8-33 13 99, E-mail: yihai.cao@ki.se
*Alternative correspondence: Yun Zhang, M.D., Ph.D., F.A.C.C., F.E.S.C., F.A.S.E., Key Laboratory of Cardiovascular Remodeling and Function Research, Chinese Ministry of Education and Chinese Ministry of Public Health, Shandong University

Qilu Hospital, Jinan, Shandong 250012, China, Tel:(+86)-531-82169139, Fax: (+86)-531-86169356, E-mail: zhangyun@sdu.edu.cn

Supplemental Table 1 and 2 were independent excel files.

Supplemental Table 3. qPCR primers

**Gene name**	**Forward primer (5'-3')**	**Reverse primer (5'-3')**
Ucp1	AGGCTTCCAGTACCATTAGGT	CTGAGTGAGGCAAAGCTGATTT
Dio2	AATTATGCCTCGGAGAAGACCG	GGCAGTTGCCTAGTGAAAGGT
Cidea	TGACATTCATGGGATTGCAGAC	GGCCAGTTGTGATGACTAAGAC
Cox7a1	GCTCTGGTCCGGTCTTTTAGC	GTACTGGGAGGTCATTGTCGG
Pgc1α	TATGGAGTGACATAGAGTGTGCT	CCACTTCAATCCACCCAGAAAG
Prdm16	CCACCAGCGAGGACTTCAC	GGAGGACTCTCGTAGCTCGAA
Ebf2	GGGATTCAAGATACGCTAGGAAG	GGAGGTTGCTTTTCAAAATGGG
Leptin	GAGACCCCTGTGTCGGTTC	CTGCGTGTGTGAAATGTCATTG
Adiponectin	GTTCCCAATGTACCCATTCGC	TGTTGCAGTAGAACTTGCCAG
Resistin	AAGAACCTTTCATTTCCCCTCCT	GTCCAGCAATTTAAGCCAATGTT
Gapdh	AGGTCGGTGTGAACGGATTTG	TGTAGACCATGTAGTTGAGGTCA

Supplemental Figure Legends

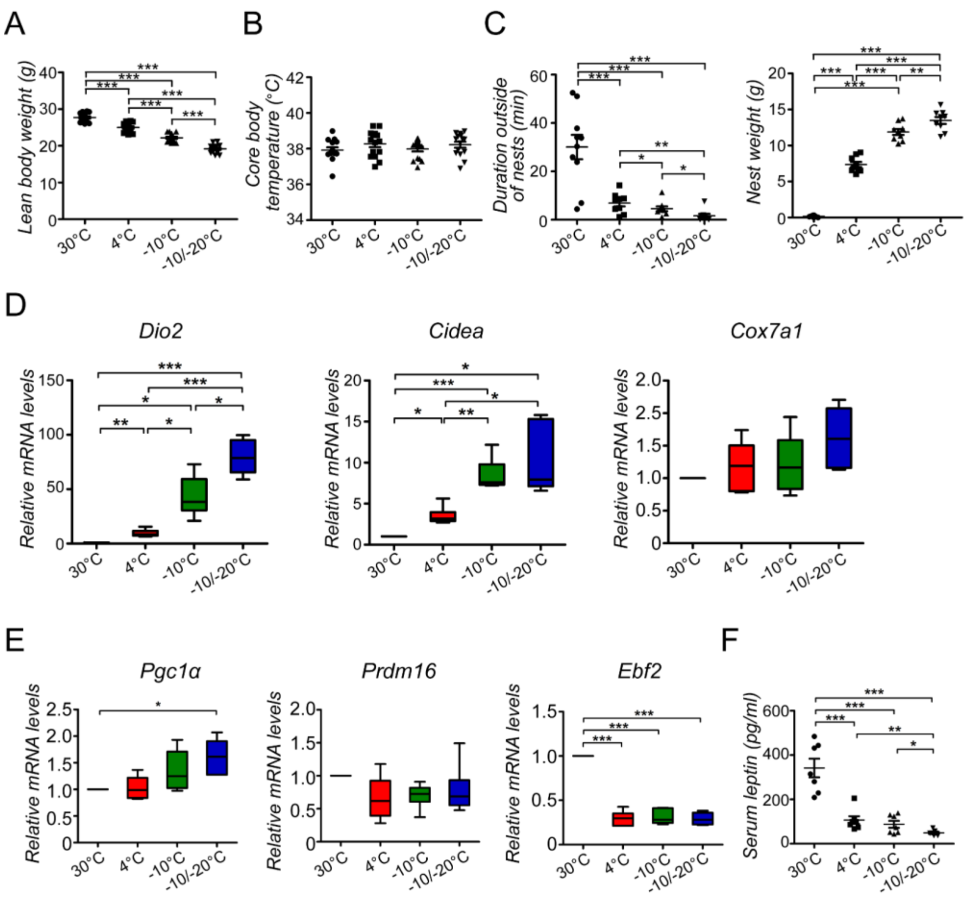

Supplemental Figure 1

Supplemental Figure 1. Physical activity and gene expression under various temperatures

A. Lean body weight without eWAT, subcWAT, rWAT, mWAT and BAT (n = 15 mice per group, data represent mean ± SEM).

B. Core body temperature under various temperatures (n = 20 mice per group, data represent mean ± SEM).

C. Out-nest time length (n = 10 mice for 30°C, n = 9 mice for 4°C, n = 9 mice for -10°C, n = 8 mice for -10/-20°C) and nest weight of mice exposure to various temperatures. (n = 10 mice for 30°C, n = 9 mice for 4°C, n = 9 mice for -10°C, n = 9 mice for -10/-20°C). Data represent mean ± SEM

D-E. qPCR analysis of mRNA levels of *Dio2*, *Cidea*, *Cox7a1*, *Pgc1α*, *Prdm16* and *Ebf2*. (n = 6 samples per groups).

F. ELISA of serum leptin levels (n = 7 samples per group, data represent mean ± SEM). * $p < 0.05$; ** $p < 0.01$; *** $p < 0.001$.

One-way ANOVA. Box-and-whiskers plots show median (line within box), upper and lower quartile (bounds of box), and minimum and maximum values (bars).

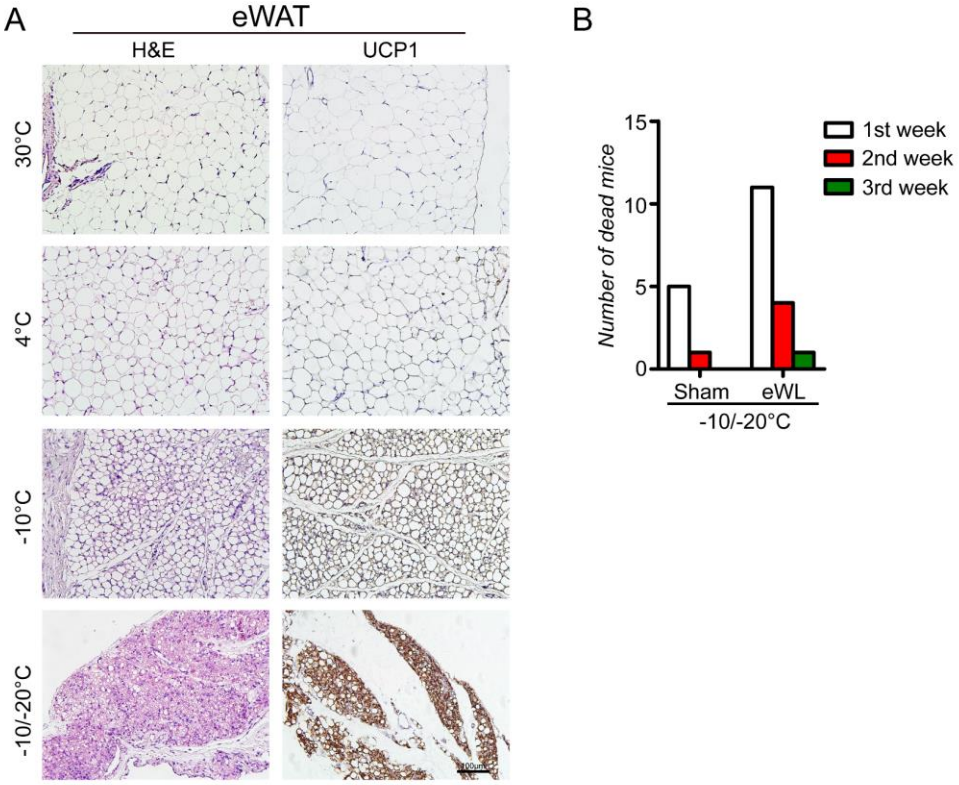

Supplemental Figure 2

Supplemental Figure 2. Overview of visceral fat browning and mouse death rate without eWAT

A. Low magnification images of H&E- and UCP-1-stained eWAT

B. Death numbers of mice without eWAT during an extreme low temperature

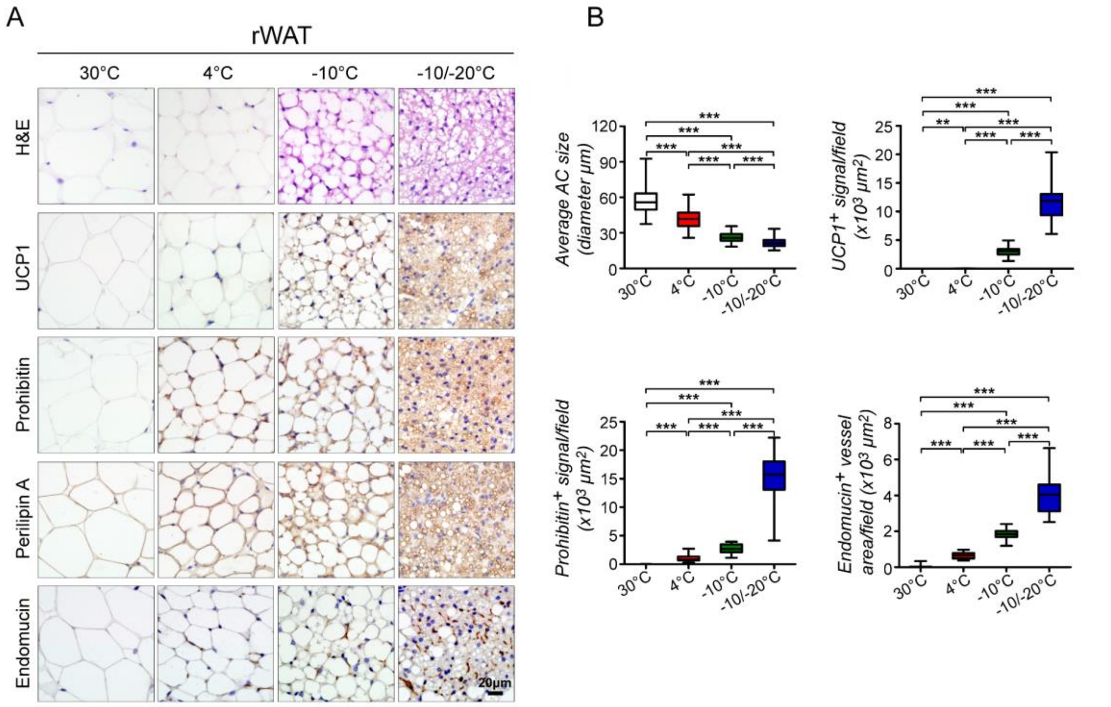

Supplemental Figure 3

Supplemental Figure 3. Browning of retroperitoneal white adipose tissue (rWAT) under various low temperature conditions

A. H&E, UCP1, prohibitin, perilipin A, and endomucin staining of retroperitoneal WAT exposed to various temperatures. Bar = 20 µm.

B. Quantification of adipocyte (AC) size, UCP1-, prohibitin-, and endomucin-positive signals of retroperitoneal WAT (30 random fields from 6 mice in each group). ** $p < 0.01$; *** $p < 0.001$.

One-way ANOVA. Box-and-whiskers plots show median (line within box), upper and lower quartile (bounds of box), and minimum and maximum values (bars).

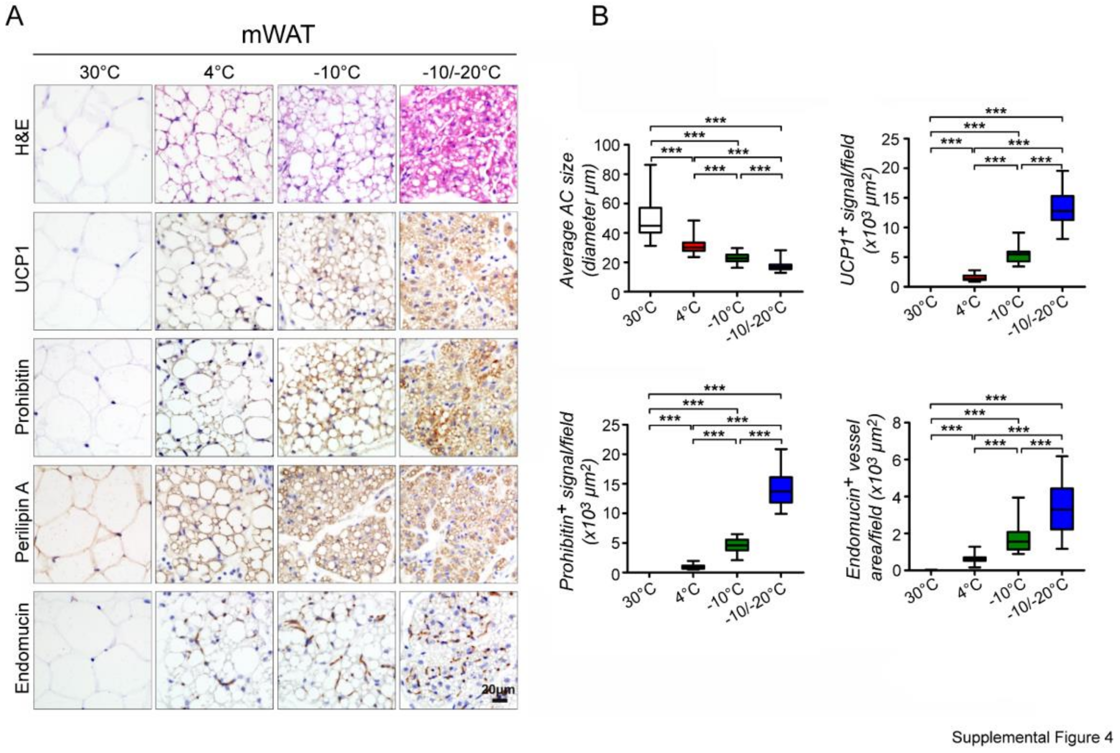

Supplemental Figure 4. Browning of mesenteric white adipose tissue (mWAT) under various low temperatures in mice

A. H&E, UCP1, prohibitin, perilipin A, and endomucin staining of mesenteric WAT exposed to various temperatures. Bar = 20 µm.

B. Quantification of AC size, UCP1-, prohibitin-, and endomucin- positive signals of mesenteric WAT (30 random fields from 6 mice in each group). ** $p < 0.01$; *** $p < 0.001$. One-way ANOVA. Box-and-whiskers plots show median (line within box), upper and lower quartile (bounds of box), and minimum and maximum values (bars).

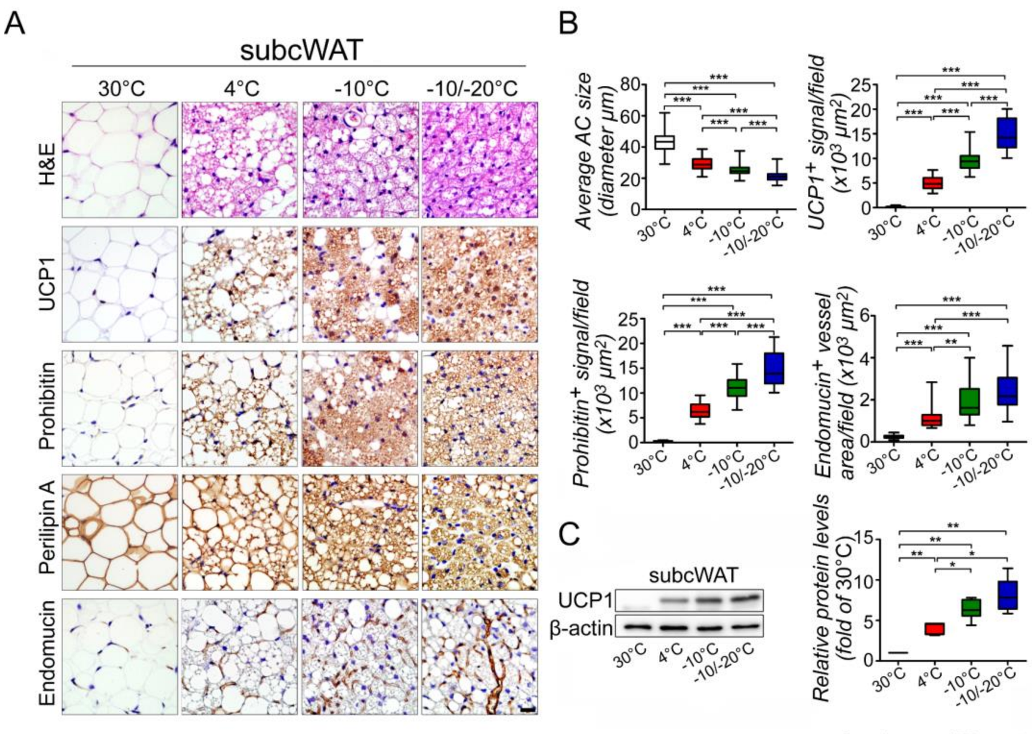

Supplemental Figure 5. Browning of subcutaneous white adipose tissue (subcWAT) under various low temperatures

A. H&E, UCP1, prohibitin, perilipin A, and endomucin staining of subcutaneous WAT exposed to various temperatures. Bar = 20 μm.

B. Quantification of AC size, UCP1-, prohibitin-, and endomucin- positive signals of subcutaneous WAT (30 random fields from 6 mice in each group). * p < 0.05; ** p < 0.01; *** p < 0.001.

C. Western blot analysis of UCP1 expressionin subcutaneous WAT (n = 6 samples per group). * p < 0.05; ** p < 0.01.

One-way ANOVA. Box-and-whiskers plots show median (line within box), upper and lower quartile (bounds of box), and minimum and maximum values (bars).

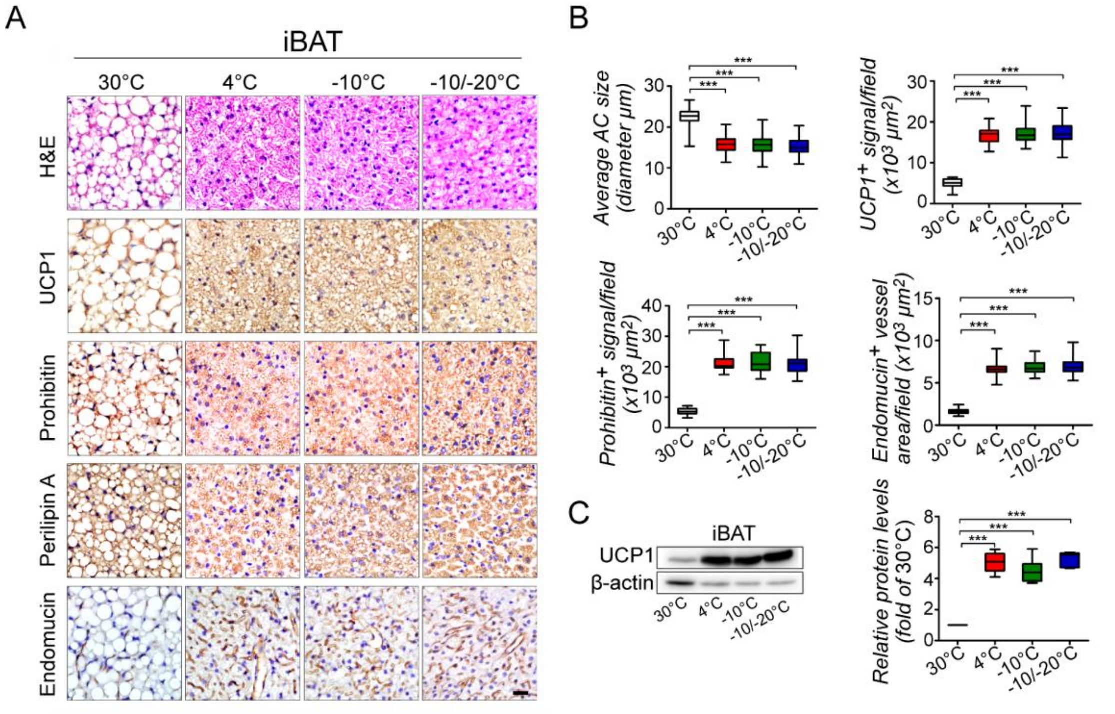

Supplemental Figure 6

Supplemental Figure 6. Activation of interscapular brown adipose tissue (iBAT) under various low temperatures

A. H&E, UCP1, prohibitin, perilipin A, and endomucin staining of iBAT exposed to various temperatures. Bar = 20 μm.

B. Quantification of AC size, UCP1-, prohibitin-, and endomucin- positive signals of iBAT (30 random fields from 6 mice in each group). *** $p < 0.001$.

C. Western blot analysis of UCP1 expression of iBAT (n = 6 samples per group). *** $p < 0.01$.

One-way ANOVA. Box-and-whiskers plots show median (line within box), upper and lower quartile (bounds of box), and minimum and maximum values (bars).

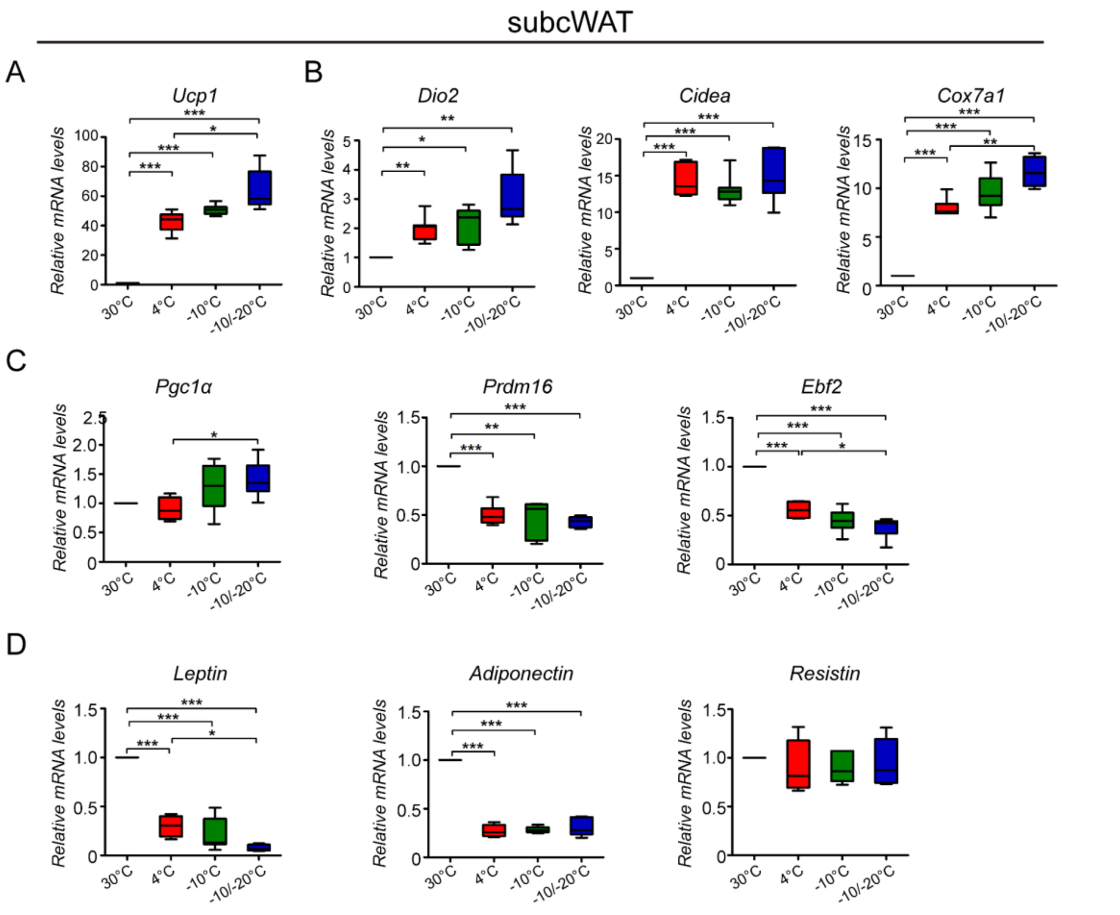

Supplemental Figure 7

Supplemental Figure 7. qPCR analysis of browning-related genes in various temperature-exposed subcutaneous WAT

A. Quantification of *Ucp1* mRNA levels in subcutaneous WAT exposed to various temperatures (n = 6 samples per group). * $p < 0.05$; *** $p < 0.001$.

B-D. Quantification of *Dio2*, *Cidea*, *Cox7a1*, *Pgc1α*, *Prdm16*, *Ebf2*, *Leptin*, *Adiponectin*, and *Resistin* mRNA levels in subcutaneous WAT exposed to various temperatures (n = 6 samples per group). * $p < 0.05$; ** $p < 0.01$; *** $p < 0.001$. One-way ANOVA. Box-and-whiskers plots show median (line within box), upper and lower quartile (bounds of box), and minimum and maximum values (bars).

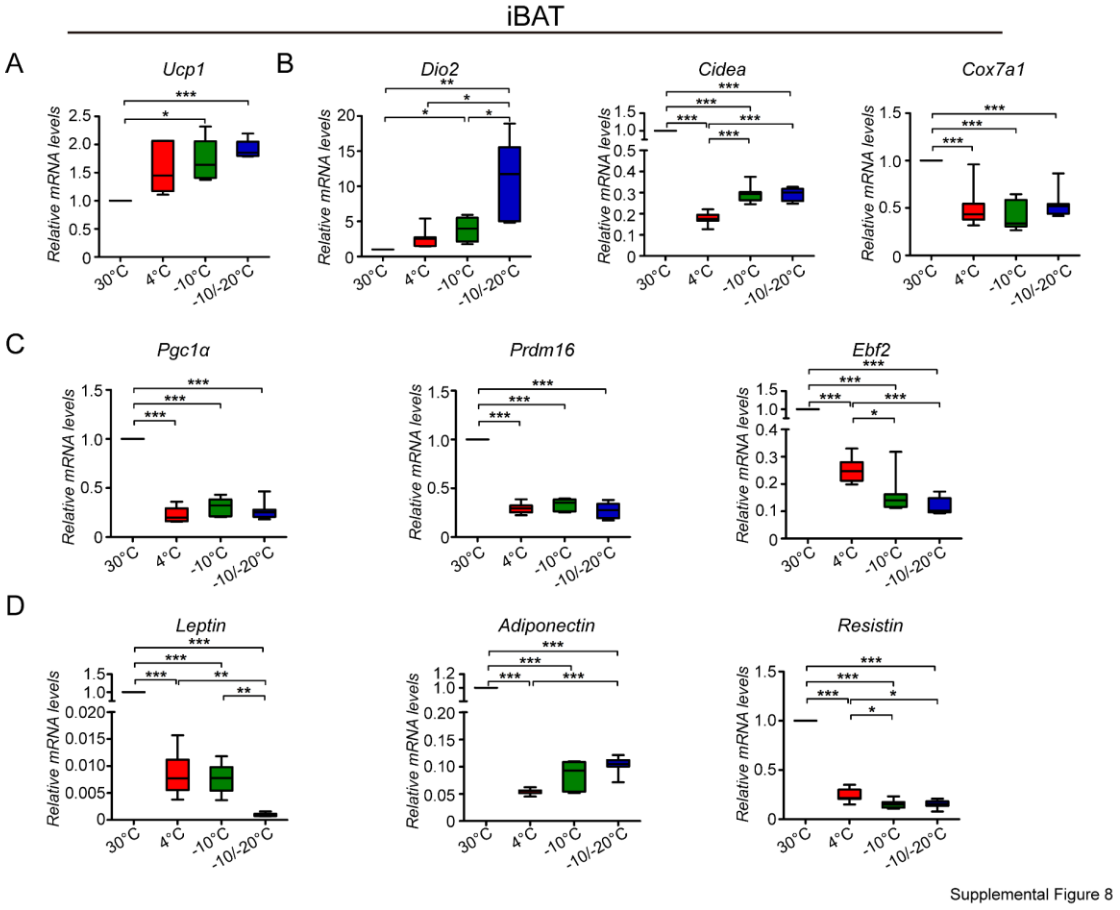

Supplemental Figure 8. qPCR analysis of browning-related genes in various temperature-exposed iBAT

A. Quantification of *Ucp1* mRNA levels in iBAT exposed to various temperatures (n = 6 samples per group). * $p < 0.05$; *** $p < 0.001$.

B-D. Quantification of *Dio2*, *Cidea*, *Cox7a1*, *Pgc1α*, *Prdm16*, *Ebf2*, *Leptin*, *Adiponectin*, and *Resistin* mRNA levels in iBAT exposed to various temperatures (n = 6 samples per group). * $p < 0.05$; ** $p < 0.01$; *** $p < 0.001$.

One-way ANOVA. Box-and-whiskers plots show median (line within box), upper and lower quartile (bounds of box), and minimum and maximum values (bars).

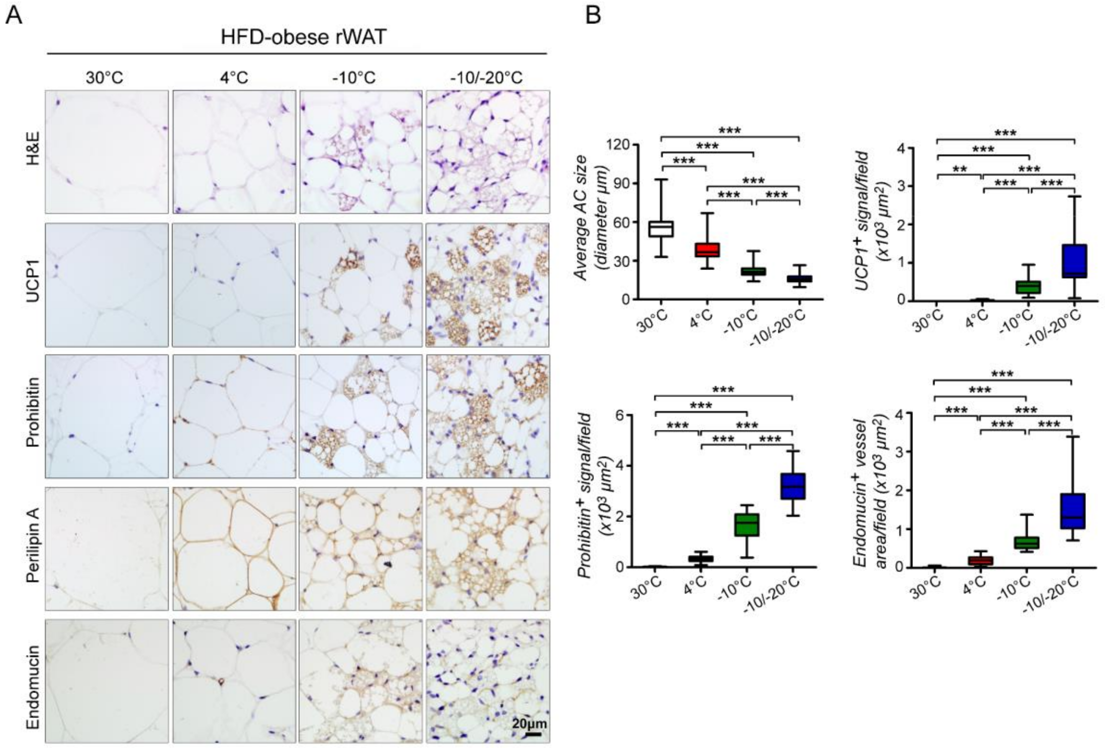

Supplemental Figure 9

Supplemental Figure 9. Browning of retroperitoneal WAT (rWAT) in HFD-induced obese mice under various low temperatures

A. H&E, UCP1, prohibitin, perilipin A, and endomucin staining of obese retroperitoneal WAT exposed to various temperatures. Bar = 20 μm.

B. Quantification of AC size, UCP1-, prohibitin-, and endomucin- positive signals in obese retroperitoneal WAT (30 random fields from 6 mice in each group). ** $p < 0.01$; *** $p < 0.001$.

One-way ANOVA. Box-and-whiskers plots show median (line within box), upper and lower quartile (bounds of box), and minimum and maximum values (bars).

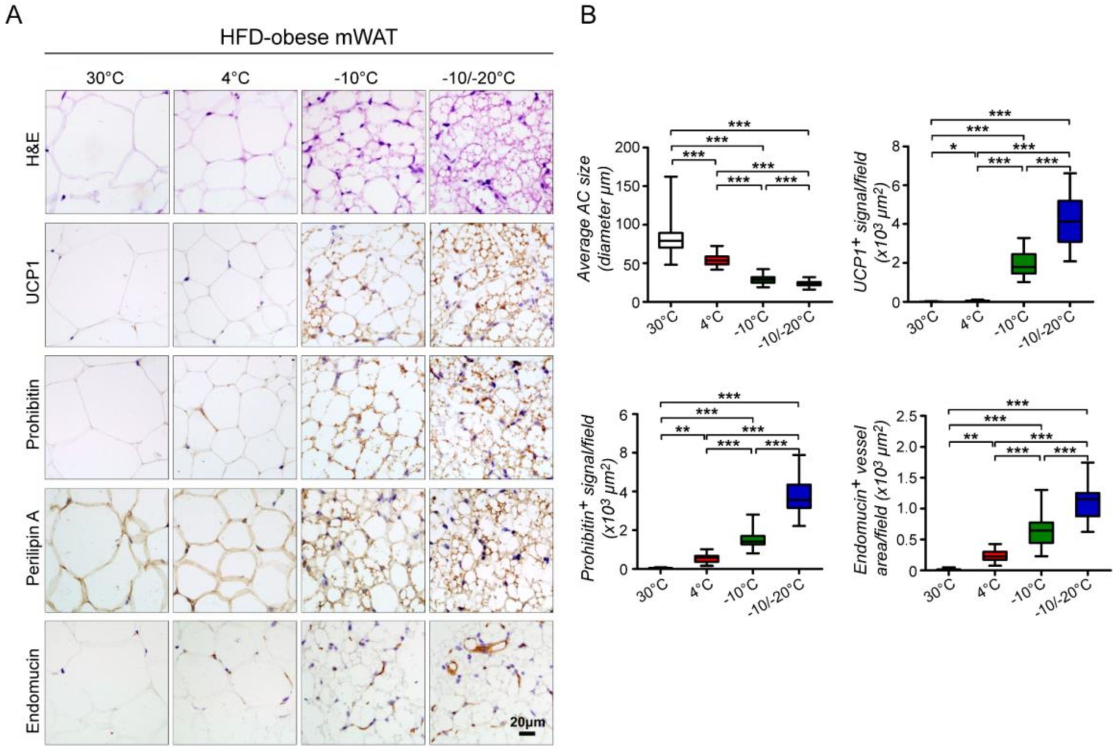

Supplemental Figure 10. Browning of mesenteric WAT (mWAT) of HFD-induced obese mice under various low temperatures

A. H&E, UCP1, prohibitin, perilipin A, and endomucin staining of obese mesenteric WAT exposed to various temperatures. Bar = 20 μm.

B. Quantification of AC size, UCP1-, prohibitin-, and endomucin-positive signals in obese mesenteric WAT (30 random fields from 6 mice in each group). * $p < 0.05$; ** $p < 0.01$; *** $p < 0.001$.

One-way ANOVA. Box-and-whiskers plots show median (line within box), upper and lower quartile (bounds of box), and minimum and maximum values (bars).

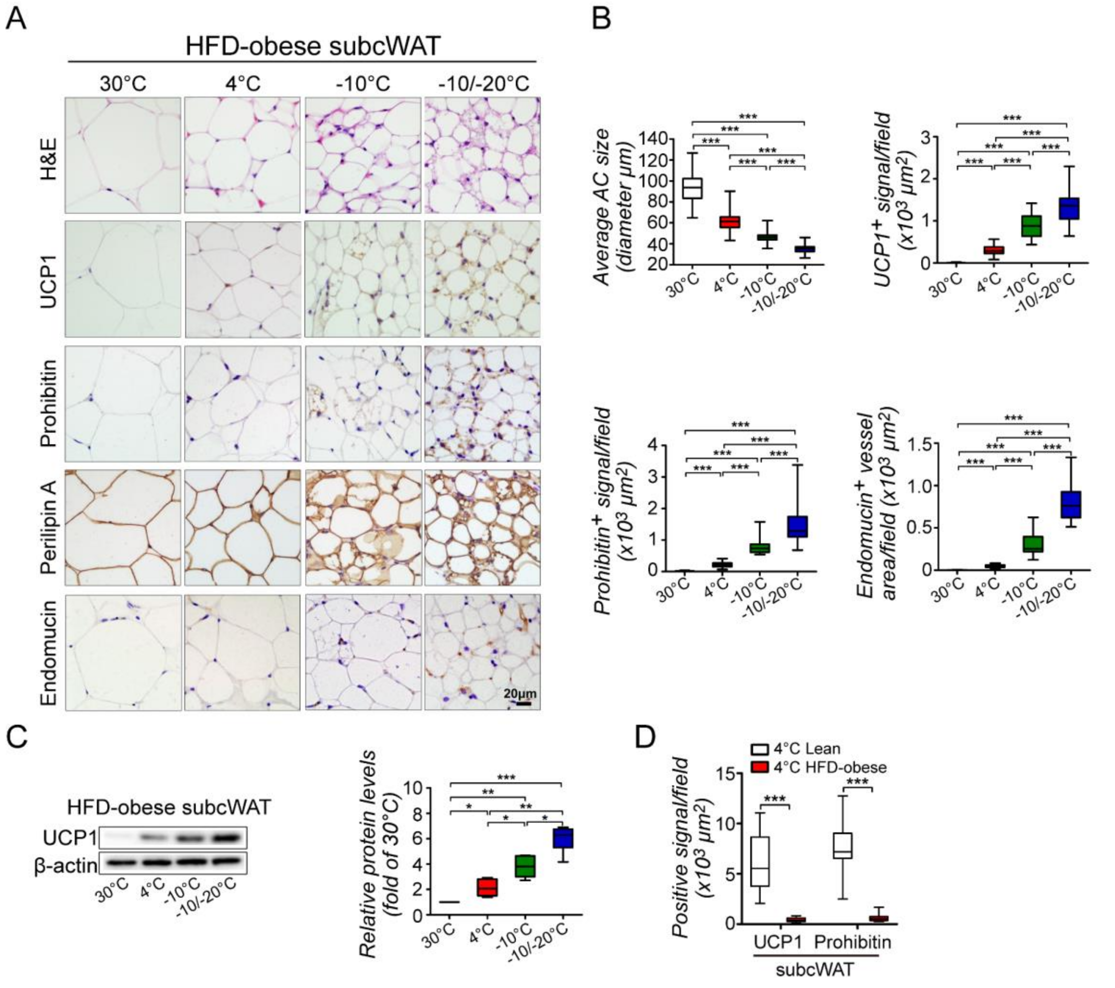

Supplemental Figure 11

Supplemental Figure 11. Browning of subcutaneous WAT of HFD-induced obese mice under various low temperature conditions

A. H&E, UCP1, prohibitin, perilipin A, and endomucin staining of obese subcutaneous WAT exposed to various temperatures. Bar = 20 µm.

B. Quantification of AC size, UCP1-, prohibitin-, and endomucin- positive signals in obese subcutaneous WAT (30 random fields from 6 mice in each group). * $p < 0.05$; ** $p < 0.01$; *** $p < 0.001$.

C. Western blot analysis of UCP1 expression in subcutaneous WAT of various groups (n = 6 samples per group). * $p < 0.05$; ** $p < 0.01$.

D. Quantification of UCP1- and prohibitin-positive signals of 4°C-exposed subcutaneous WAT of lean and obese mice (20 random fields from 6 mice in each group). *** $p < 0.001$, 2-tailed t-test.

One-way ANOVA. Box-and-whiskers plots show median (line within box), upper and lower quartile (bounds of box), and minimum and maximum values (bars).

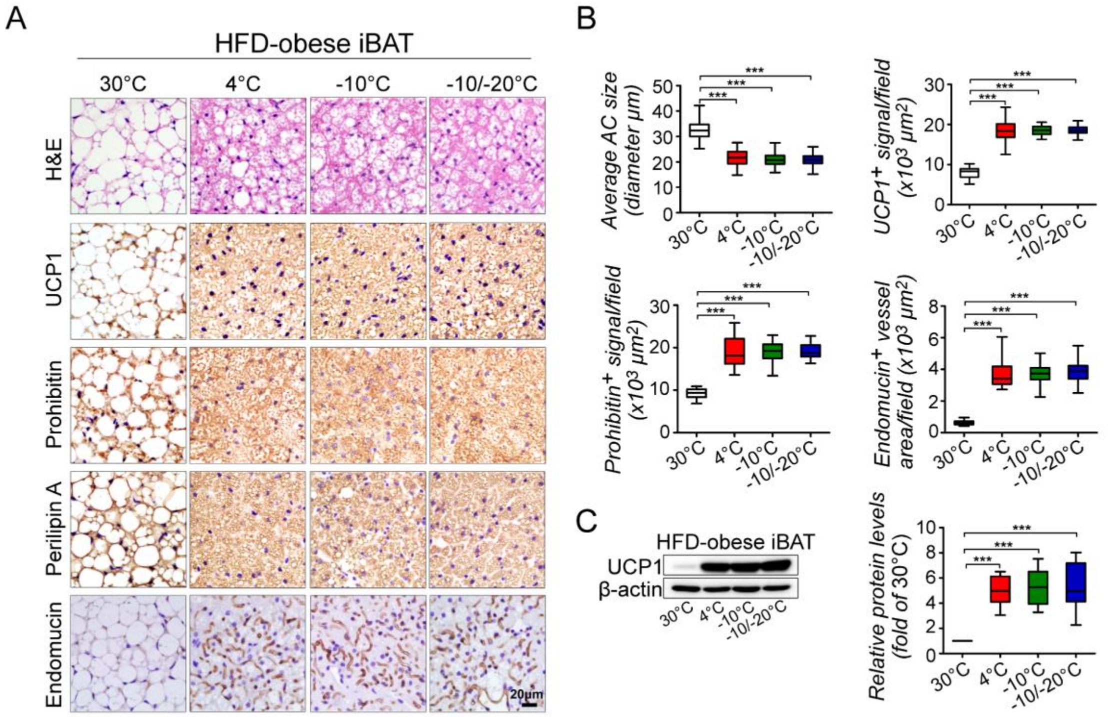

Supplemental Figure 12

Supplemental Figure 12. Activation of iBAT in HFD-induced obese mice under various low temperature conditions

A. H&E, UCP1, prohibitin, perilipin A, and endomucin staining of iBAT in obese mice exposed to various temperatures. Bar = 20 μm.

B. Quantification of obese AC size, UCP1-, prohibitin-, and endomucin- positive signals in iBAT (30 random fields from 6 mice in each group). *** $p < 0.001$.

C. Western blot analysis of UCP1 expression in iBAT of various groups (n = 6 samples per group). *** $p < 0.001$.

One-way ANOVA. Box-and-whiskers plots show median (line within box), upper and lower quartile (bounds of box), and minimum and maximum values (bars).

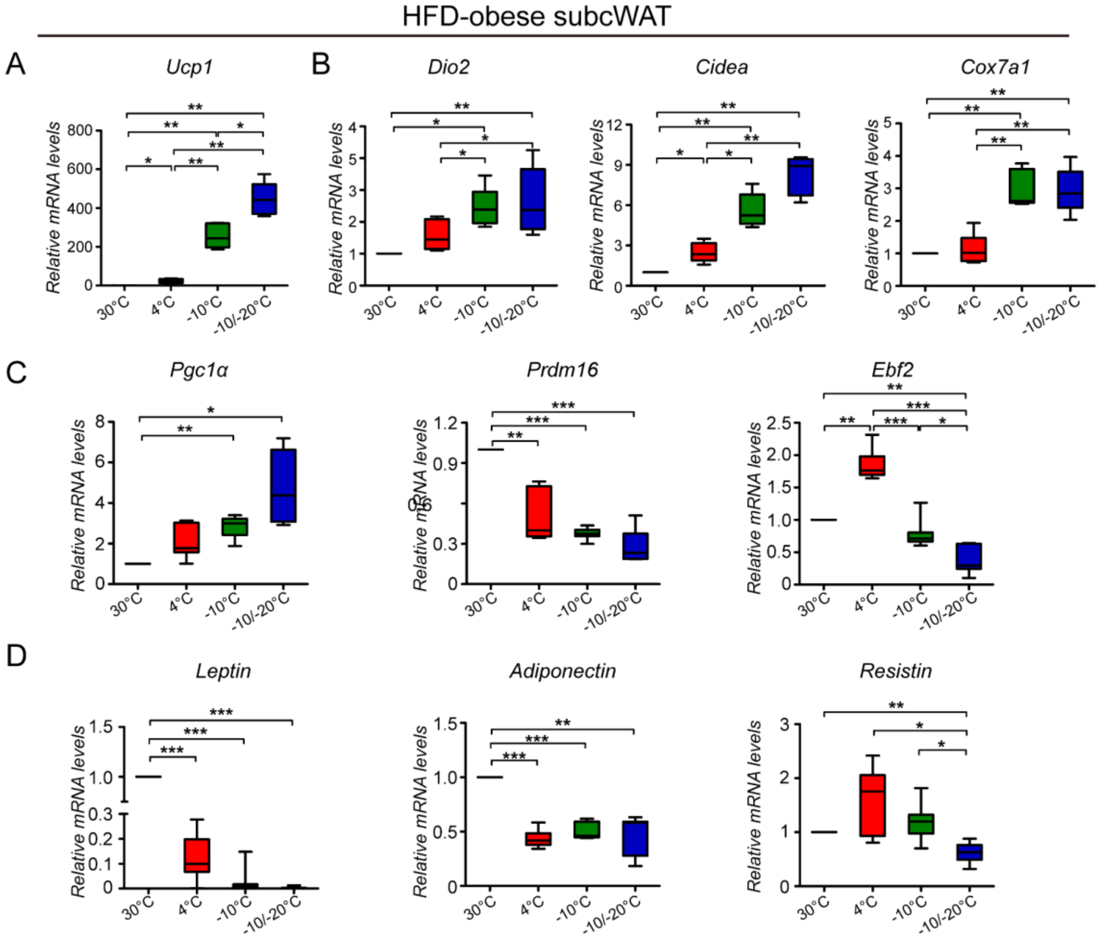

Supplemental Figure 13

Supplemental Figure 13. qPCR analysis of browning-related genes in various temperature-exposed subcutaneous WAT of HFD-induced obese mice

A. Quantification of *Ucp1* mRNA levels in subcutaneous WAT from obese mice exposed to various temperatures (6 samples per group). * $p < 0.05$; ** $p < 0.01$.

B. Quantification of *Dio2*, *Cidea*, *Cox7a1*, *Pgc1α*, *Prdm16*, *Ebf2*, *Leptin*, *Adiponectin*, and *Resistin* mRNA expression levels in subcutaneous WAT exposed to various temperatures (6 samples per group). * $p < 0.05$; ** $p < 0.01$; *** $p < 0.001$.

One-way ANOVA. Box-and-whiskers plots show median (line within box), upper and lower quartile (bounds of box), and minimum and maximum values (bars).

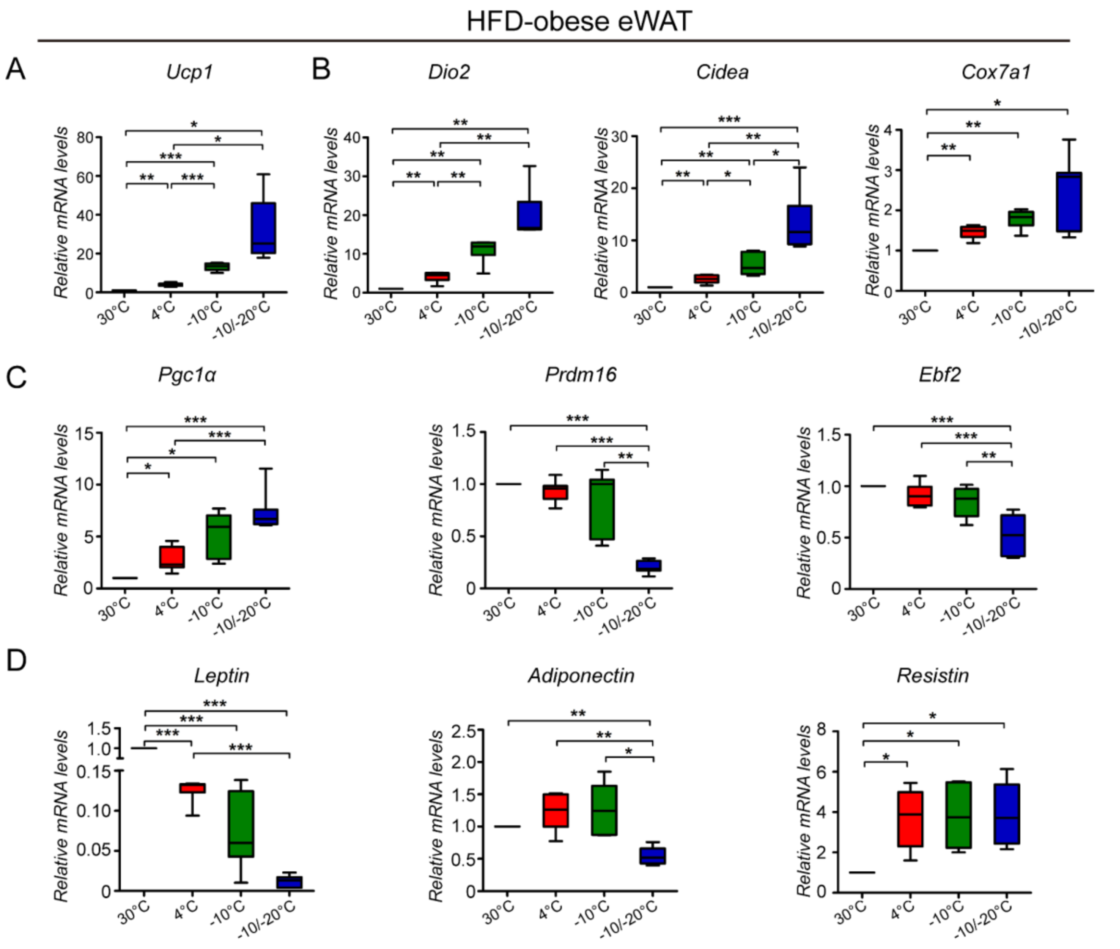

Supplemental Figure 14

Supplemental Figure 14. qPCR analyses of eWAT from HFD-induced obese animals under various temperature conditions

A. qPCR measurement of *Ucp1* levels in obese eWAT exposed to various temperatures (n = 6 samples per group). * $p < 0.05$; ** $p < 0.01$; *** $p < 0.001$.

B-D. qPCR analysis of mRNA levels of *Dio2*, *Cidea*, *Cox7a1*, *Pgc1α*, *Prdm16*, *Ebf2*, *Leptin*, *Adiponectin*, and *Resistin* in obese eWAT (n = 6 samples per group). * $p < 0.05$; ** $p < 0.01$; *** $p < 0.001$.

One-way ANOVA. Box-and-whiskers plots show median (line within box), upper and lower quartile (bounds of box), and minimum and maximum values (bars).
